# A method for improving the quality of grass carp (*Ctenopharyngodon idellus*): A comprehensive evaluation of clear water depuration based on sensory and nutritional aspects

**DOI:** 10.1016/j.fochx.2025.102601

**Published:** 2025-05-29

**Authors:** Xinyang Li, Chenyang Zhao, Lin Xu, Yuxiang Wang, Jin Yu, Xudong Weng, Ting Ye, Xiaoguo Ying, Yang Gao

**Affiliations:** a Zhejiang Provincial Key Laboratory of Health Risk Factors for Seafood, Collaborative Innovation Center of Seafood Deep Processing, College of Food and Pharmacy, Zhejiang Ocean University, Zhoushan, China; b College of Fishery, Zhejiang Ocean University, Zhoushan, China; c Longyou Aquaculture Development Center, Agricultural and Rural Bureau of Longyou County, Quzhou, China; d Zhejiang Yulaoda Agricultural Technology Co., Ltd., Quzhou, China; e Quzhou Aquatic Technology Extension Centre, Quzhou, China

**Keywords:** Fasting, Muscle texture, Flavor, Fatty acid, Amino acid

## Abstract

This study investigates the effects of clear water depuration farming on grass carp muscle quality, nutrition, and flavor. Results show that clear water depuration significantly reduces off-flavor compounds while improving muscle texture and sensory traits. After three months, fish muscle hardness, chewiness, and whiteness increased, while drip and freezing loss decreased. Low-density farming better preserved essential amino acids and unsaturated fatty acids, enhancing nutritional value. Glycine, histidine, and alanine were the main free amino acids with elevated levels. Volatile analysis showed nonanal, hexanal, and 1-octen-3-ol levels declined with time, especially under low-density conditions. Muscle texture improvements correlated positively with monounsaturated fatty acids, protein, and collagen, and negatively with moisture, pH, and polyunsaturated fatty acids. Clear water depuration may also promote collagen synthesis. Overall, a three-month depuration under low-density conditions is optimal for improving grass carp's sensory and nutritional quality without negatively affecting texture and taste.

## Highlights

• Clear water depuration for 3 months can improve the texture of grass carp.
• Low-density farming preserves essential amino acids and unsaturated fatty acids.
• Collagen synthesis and lipid metabolism are related to texture improvement.
• 3-month low-density depuration balances sensory quality and nutritional value.

## Graphical abstract

**Figure:**
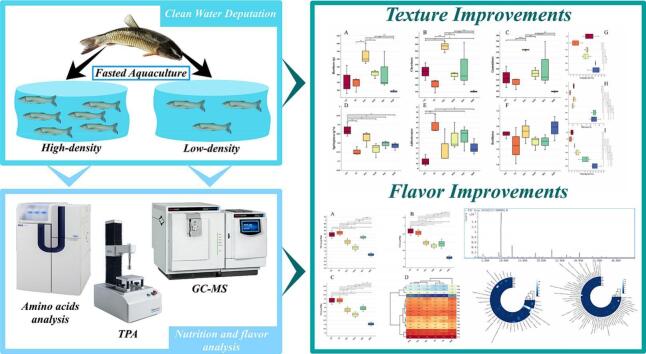

## 1 Introduction

Grass carp (*Ctenopharyngodon idellus*), a traditional freshwater aquaculture species in China, is also one of the most commercially significant farmed fish worldwide. According to The State of World Fisheries and Aquaculture 2024, grass carp currently ranks as the world's highest-yielding fish species, with an annual production exceeding 6 million metric tons (FAO, 2024). China dominates global production, contributing 5.94 million metric tons annually, as reported in the 2024 China Fishery Statistical Yearbook. Despite its high yield, grass carp suffers from low market value due to its pronounced earthy odor and loose muscle texture, which reduce consumer acceptance. Consequently, enhancing its flesh quality has become a key focus of research.

Since the 1970s, aquaculture practitioners have recognized that faba bean supplementation in feed effectively enhances the muscle structure of grass carp, particularly by improving hardness and crispness. However, this feeding strategy also induces excessive visceral lipid accumulation. Tian et al. (2019) demonstrated that faba bean-based diets suppress lipid catabolism pathways, leading to ectopic lipid deposition, which can compromise flesh flavor while increasing susceptibility to lipid oxidation. Concurrently, researchers have explored various nutritional modulation strategies to enhance fish quality. For instance, supplementation with natural submerged macrophytes has been shown to improve both the nutritional composition and textural characteristics of grass carp (Zhao et al., 2018; Zhao, Chong, Li, & Xia, 2023). Nevertheless, these approaches primarily focus on textural optimization while overlooking critical flavor attributes—specifically, the reduction of geosmin-derived earthy off-flavors and the enhancement of desirable taste compounds, such as sweet amino acids and umami-enhancing nucleotides.

To enhance the flavor quality of aquatic products, researchers have developed an innovative clear water depuration aquaculture technology. This advanced approach involves transferring fish from conventional farming systems into a recirculating aquaculture system, where they undergo controlled intermittent fasting. This process modulates metabolic pathways, significantly reducing body fat deposition while simultaneously improving muscle texture and flavor profiles (He et al., 2024). Market analyses reveal that grass carp subjected to six months of depuration can achieve a remarkable 300 % increase in commercial value compared to those raised through traditional farming methods. A growing body of evidence supports the effectiveness of this technology in optimizing the nutritional composition of fish (Shi, Li, & Liu, 2021; Yang, Shi, Qu, Qin, & Wang, 2020; Zou et al., 2023). To investigate the underlying mechanisms, Gabriel Kuniyoshi et al. (2019) conducted a proteomic study on *Piaractus mesopotamicus* after fasting. The results indicated that fasting promotes metabolic remodeling, primarily reflected in a shift from glycolytic metabolism to oxidative metabolism. Su et al. (2025) explained the degradation pathways of off-flavor compounds from the perspective of gut microbiota, suggesting that Bacteroides and Akkermansia contribute to fatty acid synthesis and the degradation of off-flavor compounds. Despite these advances, systematic evaluations of key quality parameters—including textural attributes, nutritional components, and both volatile and non-volatile flavor compounds—remain limited in current depuration studies.

To address this research gap, this study divided the fish into six groups: non-depuration fish (CK), one-month depuration (D1), three-month depuration under low and high densities (D3L and D3H), and six-month depuration under low and high densities (D6L and D6H). Through a six-month clear water depuration experiment, we systematically analyzed the effects of aquaculture mode transition and farming density on the muscle textural properties, nutritional quality, and volatile/non-volatile flavor compounds of grass carp. The findings aim to provide a theoretical foundation for muscle quality improvement and the development of high-value aquaculture technologies for grass carp.

## 2 Materials and methods

### 2.1 Fish sample preparation

The experimental grass carp specimens were purchased from the agricultural product market in Quzhou City (Zhejiang Province, China), with an initial average body mass of 1182.22 ± 162.17 g and body length of 39.31 ± 1.84 cm. The experiment was conducted in a recirculating aquaculture system at Kaihua Shiyidu Clearwater Fish Co., Ltd. (Quzhou City, China), spanning from September 3, 2024, to March 3, 2025.

A phased density regulation protocol was implemented in the study. During the initial 30-day acclimation period, all fish were cultured at a uniform density of 22.07 ± 0.25 kg/m^3^. Afterward, they were divided into two groups: a low-density group (18.43 ± 0.11 kg/m^3^) and a high-density group (25.48 ± 0.18 kg/m^3^) for continued clear water depuration. The fish were subjected to complete feed deprivation throughout the experiment, and samples were collected on day 30 (October 3, 2024), day 90 (December 3, 2024), and day 180 (March 3, 2025) of the trial period.

Aquaculture water quality parameters were continuously monitored, yielding the following results: water temperature, 21.74 ± 5.91 °C; pH, 7.42 ± 0.66; dissolved oxygen, 7.45 ± 1.42 mg/L; ammonia nitrogen, 0.32 ± 0.34 mg/L; and nitrite nitrogen, 0.12 ± 0.17 mg/L.

This study employed a stratified random sampling method, selecting six healthy grass carp from each experimental group. Live specimens were professionally transported via cold-chain logistics to the Zhejiang Provincial Key Laboratory of Health Risk Factors for Seafood. Euthanasia was performed using the pithing method. Intact muscle tissues were flash-frozen in liquid nitrogen and stored at −80 °C for subsequent physicochemical analyses.

### 2.2 Determination of growth characteristics

After measuring body weight (W), body length (L), body height, body width, and visceral mass (V), scales and skin were removed to obtain muscle samples. The calculation formulas for condition factor and viscerosomatic index (VSI) are as follows:

$$\mathrm{Condition Factor}=\frac{W\times 100}{L^{3}}$$

$$\mathrm{Viscerosomatic Index}=\frac{V\times 100}{W}$$

### 2.3 Determination of color

The chroma meter was calibrated using a white reference plate and zeroed with a black plate. Color parameters, including lightness (L^⁎^), red-green chromaticity (a^⁎^), and yellow-blue chromaticity (b^⁎^), were measured in both belly and dorsal muscles using a DS-210 colorimeter (Caipu Technology Co., Ltd., Taizhou, China). The whiteness index (WI) was calculated using the following formula:

$$\text{Whiteness index}=100-\sqrt{{\left(100-L^{*}\right)}^{2}+{a^{*}}^{2}+{b^{*}}^{2}}$$

### 2.4 Determination of texture characteristics and water-holding capacity (WHC)

Following the method described by Han et al. (2024) with minor modifications, dorsal muscle samples were cut into 20 mm × 20 mm × 10 mm cubes. Texture profile analysis was performed using an iTexture texture analyzer (Zhejiang Keqi Instrument Equipment Co., Ltd., Hangzhou, China) with the following parameters: P50 probe; pretest speed = 2 mm/s; post-test speed = 2 mm/s; test speed = 1 mm/s; trigger force = 5 g; deformation rate = 30.00 %; dwell time = 6 s.

For cooking loss determination, 5 g muscle samples were sealed in cooking bags and immersed in a 75 °C water bath for 10 min. After thermal treatment, samples were surface-dried and weighed. Drip loss and thawing loss were measured using 5 g muscle samples stored at 4 °C and − 20 °C for 24 h, respectively. Samples were then thawed at room temperature for 30 min, surface-dried, and reweighed.

### 2.5 Determination of basic nutritional components

Samples from dorsal muscle were used for basic nutritional analysis. Moisture and ash contents were determined according to GB 5009.4-2016, crude lipid content by GB 5009.6-2016, and crude protein content following GB 5009.5-2016.

Collagen content was measured using the method described by Dong and Dai (2023) with modifications. Briefly, 3 g of dorsal muscle was hydrolyzed with HCl (1:1, 5 mL) at 110 °C for 22 h. After adjusting the volume to 10 mL, 50 μL aliquot was vacuum-dried (60 °C, 2 h), then derivatized under nitrogen protection with a freshly prepared reagent (ethanol: phenylisothiocyanate: water: triethylamine = 7:1:1:1) for 30 min at room temperature. The reaction mixture was then diluted with mobile phase A (0.1 mol/L sodium acetate-acetonitrile, 97:3, pH 6.5), filtered through a 0.45 μm membrane, and analyzed by HPLC using a C18 column (4.6 × 250 mm, 5 μm) with an injection volume of 10 μL, column temperature of 40 °C, detection wavelength of 254 nm, mobile phase A (0.1 mol/L sodium acetate-acetonitrile, 97:3) and B (acetonitrile-water, 80:20), a gradient program of 0 → 14 → 29 → 30 → 37 → 38 → 45 min (A%: 100 → 85 → 66 → 0 → 0 → 100 → 100; B%: 0 → 15 → 34 → 100 → 100 → 0 → 0), and a flow rate of 1.0 mL/min.

### 2.6 Determination of hydrolyzed amino acids and fatty acids

The determination of hydrolyzed amino acids followed the method of Lin et al. (2018) using an amino acid analyzer (LA8080, Hitachi, Japan). Briefly, 3 g of sample was hydrolyzed with 5 mL HCl (1:1) at 110 ± 1 °C for 22 h in a hydrolysis tube. After cooling, the hydrolysate was filtered and adjusted to 10 mL. A 0.05 mL aliquot was evaporated to dryness under nitrogen, reconstituted with 0.02 mol/L HCl to 2 mL, and filtered through a 0.22 μm membrane. Analysis conditions included: mobile phase flow rate 0.35 mL/min, column temperature 135 °C, reaction buffer flow rate 0.3 mL/min, detection wavelength transition from 570 nm to 440 nm, and injection volume 20 μL. Amino acid concentrations were quantified using external standard calibration curves.

Fatty acid analysis was performed according to our previous method using GC–MS (GC6890N-5975MS, Agilent, Missouri, USA) (Li et al., 2025). Fatty acid methyl esters were separated on an Agilent DB-23 capillary column (30 m × 0.25 mm × 0.25 μm) with helium carrier gas (1.0 mL/min) and the following temperature program: 130 °C (1 min) → 5 °C /min → 220 °C (5 min). The injector and detector temperatures were 220 °C, with a split ratio of 5:1 and injection volume of 0.2 μL. MS parameters included: ion source and quadrupole temperatures 200 °C, scanning range 33–500 *m*/*z*, and ionization voltage 70 eV. Identification was achieved via NIST library matching (similarity ≥90 %), and quantification used the peak area normalization method.

### 2.7 Determination of free amino acids (FAAs)

The analysis of free amino acids was performed according to the method described by Han et al. (2024). Briefly, 3 g of fish dorsal muscle was homogenized with 15 mL of 3 % trichloroacetic acid solution in a 50 mL centrifuge tube for 2 min, followed by centrifugation at 10,000*g* and 4 °C for 10 min to obtain the supernatant. The supernatant was then filtered through a 0.45 μm membrane prior to analysis using an automated amino acid analyzer (LA8080, Hitachi, Tokyo, Japan).

### 2.8 Determination of volatile components and relative odor activity value (ROAV)

The analysis of volatile compounds was performed according to our previously established HS-SPME-GC-MS method using a 6890N-5975B GC-MS system (Agilent Technologies, USA) equipped with an HP-5MS capillary column (30 m × 0.25 mm × 0.25 μm, 0.25 μm film thickness) (Wu et al., 2025; Ying et al., 2024).

A 3 g fish muscle sample was weighed into a 20 mL headspace vial containing 2 mL saturated sodium chloride solution and spiked with 2-octanol internal standard (final concentration: 3 mg/L). The vial was equilibrated at 80 °C for 30 min, followed by headspace extraction using a SPME fiber (50/30 μm, DVB/Carboxen™/PDMS StableFlex™) for an additional 30 min at 80 °C. The fiber was then desorbed in the GC injection port at 250 °C for 5 min. GC separation was performed on an HP-5MS column (30 m × 0.25 mm × 0.25 μm) with the following temperature program: 50 °C (2 min), ramped to 180 °C at 5 °C/min (5 min hold), then to 250 °C at 10 °C/min (5 min hold). MS detection was conducted with the transfer line at 280 °C, ion source at 230 °C, and quadrupole at 150 °C, using electron impact ionization (70 eV) in full scan mode (*m*/*z* 40–600). Volatile compounds were identified by matching mass spectra to NIST/Wiley libraries (match factor ≥ 80 %) and comparing calculated linear retention indices (based on C₈-C₂₀ n-alkanes) with reference standards.

The ROAV is calculated with reference to Zhu, Chen, Chen, Chen, and Deng (2020) as shown below:

$$\text{ROAV}=\frac{C_{i}}{C_{t}}\times \frac{T_{t}}{T_{i}}\times 100\%$$

where Ci and Ti correspond to the relative content (%) and odor threshold (mg/kg) of each volatile flavorant, and Ct and Tt correspond to the relative content (%) and odor threshold (mg/kg) of the volatile flavorant that contributes most to the overall flavor, respectively. The threshold values of volatile compounds were referenced from Van Gemert (2011).

### 2.9 Statistical analysis

All experimental data were collected in triplicate and analyzed using SPSS 27 (IBM Corp., Chicago, IL, USA) to calculate mean values ± standard deviation. Statistical significance was assessed through one-way analysis of variance (ANOVA) with a significance threshold of *P* < 0.05. Pearson correlation analysis, implemented in R (v4.3.1, R Foundation), was employed to evaluate relationships between sensory attributes and nutritional quality parameters. Visualization of GC–MS metabolite clustering patterns and correlation heatmaps was performed using Chiplot (https://www.chiplot.online/).

## 3 Results and discussion

### 3.1 Growth characteristics and basic nutrient composition

Following clear water depuration, grass carp exhibited varying degrees of body size reduction, with significant differences observed in visceral weight and the VSI (Table S1) (*p* < 0.05). The lowest VSI was recorded after three months of depuration. Given that grass carp muscle has a naturally low fat content, with lipids primarily stored in the viscera, the reduction in VSI suggests that visceral lipids were preferentially catabolized for energy production during depuration(Wu et al., 2015). This finding aligns with the observations of Liao et al. (2017). The degradation of visceral lipids was accompanied by a reduction in overall body weight. Notably, the D3L and D6H groups exhibited the highest body weights post-depuration, resulting in the highest dressing percentages. No significant differences were detected in body length, body width, or body height during the first three months of depuration; however, body height showed a significant decrease after six months. Additionally, no significant differences in the condition factor were observed among the six experimental groups.

During the depuration period, glycogen, protein, and fat in fish tissues serve as primary energy sources to sustain normal metabolism. Previous studies have shown that hepatic glycogen is typically the first energy substrate mobilized during early starvation, with fat mobilization occurring almost simultaneously following hepatic glycogen depletion. When glycogen and fat reserves are nearly exhausted, protein is subsequently utilized for energy (Ren et al., 2025). In this study, both fat and protein content in fish muscle exhibited a negative correlation with depuration duration, with fat decreasing more significantly than protein. This suggests that fat serves as the preferred energy source during the depuration process. The moisture content, pH, and ash content of fish muscle initially declined before increasing, reaching peak levels after six months of depuration (Table S2). Collagen content, however, peaked after three months of depuration. Notably, the effects of culture density on fish muscle composition were primarily observed in the D3L and D3H groups, whereas its influence became negligible after six months.

### 3.2 Color

**Fig. 1:**
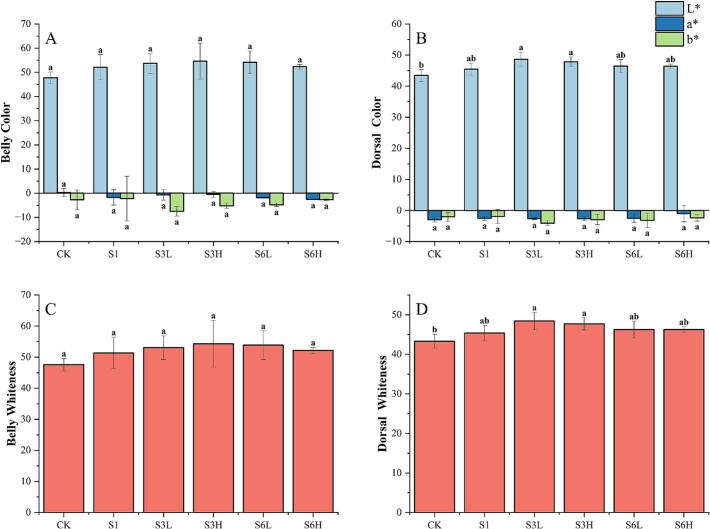
Effect of depuration on fish muscle color. A and B are the L^⁎^, a^⁎^, and b^⁎^ of the belly and dorsal muscles, respectively, and C and D are the WI of the belly and dorsal muscles, respectively.

To comprehensively assess color changes, we analyzed the color differences between the belly and dorsal muscles (Fig. 1). Among the measured parameters, L^⁎^ exhibited a positive correlation with the duration of clear water depuration, whereas a^⁎^ and b^⁎^ showed negative correlations. The trend in whiteness variation aligned with that of L^⁎^, indicating that the fish flesh gradually became whiter. These findings suggest that clear water depuration enhances the sensory attributes of grass carp. Our results are consistent with the findings of Bjørnevik et al. (2017), who reported that fasting environments can influence fish flesh color by modulating pH levels, with pH being negatively correlated with L^⁎^. The impact of farming density on color varied between different muscle sections. Notably, L^⁎^ and WI peaked after three months of depuration, with high-density farming yielding a higher WI in belly muscle, whereas low-density farming resulted in whiter dorsal muscles. Given that the dorsal muscle is the primary edible portion of grass carp, low-density farming is more favorable for optimizing flesh color.

### 3.3 Texture and WHC

**Fig. 2:**
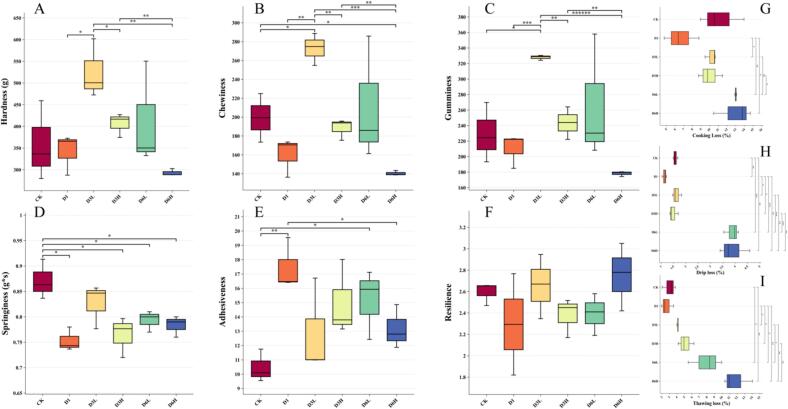
Effect of depuration on fish muscle texture and WHC. Figs. A-F show textural characteristics and Figs. G-I show water holding capacity.

Texture is a crucial indicator of sensory quality and is widely associated with increased hardness and chewiness. Following clear water depuration, the hardness, chewiness, and adhesiveness of fish muscle significantly improved, with the effects being more pronounced under low-density farming conditions. After three months of low-density clear water depuration, the hardness and chewiness reached their peak, indicating that the D3L group exhibited the best texture (Fig. 2). However, when the depuration period was extended to six months, a decline in texture quality was observed. The texture of fish muscle is closely related to its nutritional composition. Studies have shown that hardness is negatively correlated with lipid content. Research by Lv, Hu, Xiong, You, and Fan (2018) indicated that the increase in fish muscle hardness was attributed to lipid depletion caused by starvation. The observed texture degradation in D6H and D6L may be due to a reduction in protein content and an increase in ash content. A study on wild European flounder also demonstrated that lower ash content correlated with higher hardness (Kendler, Yilmaz, Jakobsen, Mangor-Jensen, & Lerfall, 2024). Overall, low-density clear water depuration appears to be an effective approach for improving fish muscle texture.

WHC reflects the interaction between proteins and water in fish muscle, with higher WHC generally indicating greater juiciness. In this study, no significant differences were observed in cooking loss, whereas drip loss and thawing loss remained stable after three months but increased significantly after six months. By the six-month mark, WHC exhibited a notable decline (Fig. 2H). The rise in drip loss and thawing loss may be attributed to the elevated moisture content in D6L and D6H. Under low-temperature conditions, excessive moisture promotes the formation of ice crystals, leading to physical damage to muscle tissue (Klinmalai, Fong-in, Phongthai, & Klunklin, 2021). Additionally, numerous studies have indicated that starvation-induced changes in pH can alter WHC (Bjørnevik et al., 2017; Peng et al., 2024). However, no clear trend in pH variation was observed in this study, which may be attributed to species-specific differences. Similarly, WHC is closely linked to lipid content. Previous research suggests that a reduction in polyunsaturated fatty acids in fish muscle may contribute to WHC decline. Therefore, further analysis of fatty acid composition is warranted (Álvarez, Fontanillas, Hernández-Contreras, & Hernández, 2020).

### 3.4 Hydrolyzed amino acid composition

**Fig. 3:**
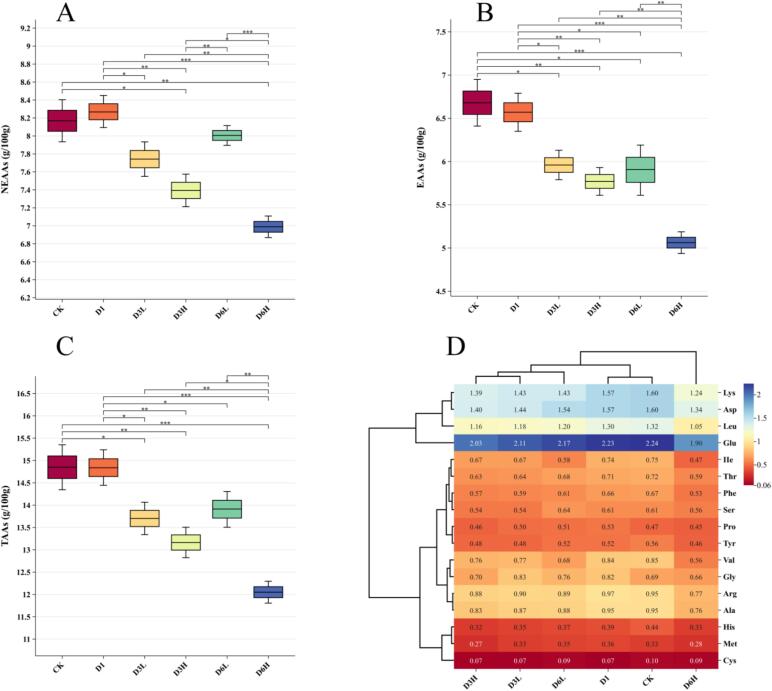
Amino acid content of different samples. Figs. A-C show the contents of NEAAs, NEAAs and TAAs, respectively, and Fig. D shows the thermogram of hydrolysed amino acid composition. Asp refers to aspartic acid, Thr to threonine, Ser to serine, Gly to glycine, Ala to alanine, Val to valine, Met to methionine, Ile to isoleucine, Leu to leucine, Tyr to tyrosine, Phe to phenylalanine, Lys to lysine, His to histidine, Glu to glutamic acid, Cys to cysteine, Arg to arginine, and Pro to proline. TAAs represent total amino acids.

The nutritional value of fish protein is determined not only by its overall content but also by its amino acid composition. In this study, 17 amino acids were detected across the four sample groups, including 8 essential amino acids (EAAs) and 9 non-essential amino acids (NEAAs) (Fig. 3D). Following clear water depuration, both EAAs and NEAAs exhibited a negative correlation with farming duration, likely due to amino acid depletion caused by starvation. Research by He, Li, Yan, and Han (2022) demonstrated that amino acids in the dorsal muscle of starved carp were consumed in the following order: His > Arg > Ile > Val > Ala > Lys > Leu > Asp > Gly. In the present study, all nine of these amino acids showed a significant decline after six months of farming, with Ile experiencing the most substantial reduction. No significant differences in EAA content were observed among D3L, D3H, and D6L, whereas D6H exhibited a significant decrease. Amino acid clustering analysis revealed that D3H, D3L, and D6L were more closely related, while D6H displayed more pronounced differences. These findings suggest that prolonged depuration leads to EAA depletion, whereas low-density farming mitigates this effect, helping to preserve the nutritional value of the fish.

### 3.5 Fatty acid composition

A total of 15 fatty acids were detected in this study, with palmitic acid (C16:0), oleic acid (C18:1n9c), and linoleic acid (C18:2n6c) being the most abundant saturated fatty acid (SFAs), monounsaturated fatty acid (MUFAs), and polyunsaturated fatty acid (PUFAs) in fish muscle, respectively (Table 1). Following clear water depuration, oleic acid content significantly increased, whereas linoleic acid showed a significant decline. This result aligns with previous studies (Xu et al., 2022; Zhan et al., 2024). Among the detected fatty acids, PUFAs content exhibited the most substantial reduction, while SFA content remained stable, and MUFAs content increased. This trend suggests that PUFAs serve as the primary energy source for grass carp metabolism. The selective utilization of fatty acids is strongly influenced by environmental factors. During overwinter starvation, grass carp preferentially metabolize MUFAs rather than PUFAs to mitigate oxidative stress caused by free radicals generated during PUFAs metabolism (Sun, Wu, & Ji, 2021). Among these, n-3 PUFAs are key lipid components in aquatic products, known for their important anti-inflammatory and anti-cancer properties (Liu et al., 2020). Clear water depuration led to a significant reduction in n-3 PUFA levels, reaching a minimum in the D6 group. However, nutrient retention in the D6L group was significantly higher than in D6H. Furthermore, consistent with the trends observed in amino acid composition, low-density farming exhibited superior nutritional value, as the UFA content under low-density conditions was significantly higher than that under high-density farming.

**Table 1:** Effect of clear water depuration on fatty acid composition of fish muscle (%).

Fatty acids	Content					
	CK	D1	D3L	D3H	D6L	D6H
C14:0	0.52 ± 0.03d	0.5 ± 0.01d	0.81 ± 0.01a	0.67 ± 0.01b	0.62 ± 0.01c	0.84 ± 0.01a
C16:0	18.98 ± 0.05b	16.49 ± 0.02f	18.46 ± 0.03c	17.46 ± 0.03e	17.78 ± 0.01d	19.16 ± 0.02a
C18:0	7.1 ± 0.01d	6.46 ± 0e	5.78 ± 0.01f	7.79 ± 0.01c	7.81 ± 0b	8.96 ± 0a
SFAs	26.6 ± 0.02b	23.45 ± 0.02f	25.06 ± 0.01e	25.92 ± 0.02d	26.21 ± 0.01c	28.96 ± 0.01a
C16:1	2.93 ± 0.02b	2.25 ± 0.01e	3.59 ± 0.01a	2.49 ± 0.01d	2.54 ± 0c	2.55 ± 0.02c
C18:1n9c	24.07 ± 0.07f	25.43 ± 0.02e	33.77 ± 0.09c	29 ± 0.08d	34.05 ± 0.01b	36.48 ± 0.04a
C20:1	0.76 ± 0.01e	0.98 ± 0.01d	1.57 ± 0.01c	1.69 ± 0.01a	1.56 ± 0.02c	1.59 ± 0.01b
C22:1n9	–	–	0.32 ± 0.01a	–	–	–
C24:1	0.95 ± 0.06d	1.19 ± 0.01c	1.37 ± 0.01a	1.26 ± 0.01b	0.38 ± 0.03e	–
MUFAs	28.71 ± 0.02e	29.85 ± 0d	40.61 ± 0.06a	34.45 ± 0.06c	38.54 ± 0.05b	40.62 ± 0.02a
C18:2n6c	22.74 ± 0.05a	29.16 ± 0.05b	20.78 ± 0.04d	20.92 ± 0.06c	19.82 ± 0.04e	19.31 ± 0.01f
C18:3n3	1.4 ± 0.01a	1.41 ± 0.03a	1.05 ± 0.01d	1.26 ± 0.03b	0.94 ± 0.01e	1.11 ± 0.01c
C20:2	1.41 ± 0.01b	1.61 ± 0.01a	1.08 ± 0.09e	1.33 ± 0.01c	1.31 ± 0.05c	1.18 ± 0.01d
C20:3n6	1.62 ± 0.02a	1.51 ± 0.01b	1.52 ± 0.02b	1.62 ± 0.06a	1.67 ± 0.03a	1.22 ± 0.01c
C20:4n6	11.13 ± 0.02a	9.47 ± 0.01c	8.01 ± 0.01e	10.39 ± 0.02b	9.3 ± 0.1d	6.26 ± 0.01f
C20:5n3	0.44 ± 0.01a	–	–	–	–	–
C22:6n3	5.96 ± 0.01a	3.54 ± 0.02c	1.9 ± 0.01e	4.1 ± 0.01b	2.22 ± 0d	1.35 ± 0.01f
PUFAs	44.69 ± 0b	46.7 ± 0.02a	34.33 ± 0.07e	39.64 ± 0.04c	35.26 ± 0.04d	30.42 ± 0.03f
UFAs	73.4 ± 0.02e	76.55 ± 0.02a	74.94 ± 0.01b	74.08 ± 0.02c	73.79 ± 0.01d	71.04 ± 0.01f

Note: Different superscript letters (a, b, c, d, e, f) indicate significant differences (P < 0.05) among different aquaculture zones. SFAs refer to saturated fatty acids, MUFAs to monounsaturated fatty acids, PUFAs to polyunsaturated fatty acids, and UFAs to unsaturated fatty acids.

### 3.6 Changes in non-volatile components

The composition and content of FAAs are key factors influencing the taste profile of fish muscle. In this study, a total of 14 FAAs were identified in grass carp samples subjected to different durations of clear water depuration (Table 2). Among these, Gly, Ala, Ser, Thr, and Arg contribute to sweetness, while His, Lys, Leu, Val, Ile, Met, Tyr, and Phe impart bitterness (Li et al., 2024). Notably, the concentration of sweet-tasting FAAs (SFAAs) was significantly higher in the low-density farming group than in the high-density group, suggesting that low-density clear water depuration enhances SFAAs content and increases total free amino acids (TFAAs) levels.

**Table 2:** Composition of free amino acids in fish muscle (mg/g).

Free amino acids	Threshold (mg/g)	Content					
		CK	D1	D3L	D3H	D6L	D6H
Gly	1.88	9.89 ± 0.33c	14.12 ± 0.38b	17.54 ± 2.05a	13.88 ± 0.49b	17.3 ± 0.67a	17.02 ± 1.1a
Ala	1.06	6.01 ± 0.15a	3.36 ± 0.13b	2.02 ± 0.2c	2.09 ± 0.08c	3.41 ± 0.37b	2.41 ± 1.71b
His	6.98	–	13.82 ± 0.96a	10.23 ± 0.5b	11.2 ± 0.25b	14.1 ± 3.52a	11.01 ± 0.72bc
Ser	2.63	0.9 ± 0.02a	0.9 ± 0.06a	0.83 ± 0.04ab	0.75 ± 0.05b	1.55 ± 0.13a	1.17 ± 0.32b
Lys	11.69	3.9 ± 0.11c	6.52 ± 0.26a	4 ± 0.12c	5.48 ± 0.52b	1.97 ± 0.36d	1.93 ± 0.65d
Thr	4.17	1.65 ± 0.11a	1.56 ± 0.04a	1.3 ± 0.03b	1.18 ± 0.05b	0.99 ± 0.17 cd	0.97 ± 0.16d
Arg	13.07	0.99 ± 0.03a	0.94 ± 0.05a	–	–	–	–
Leu	1.44	1.29 ± 0.03a	1.12 ± 0.05b	0.97 ± 0.06c	0.86 ± 0.02d	0.58 ± 0.05d	0.53 ± 0.12d
Val	3.51	0.76 ± 0.02a	0.69 ± 0.03b	0.63 ± 0.02c	0.58 ± 0.01d	0.49 ± 0.05d	0.46 ± 0.08d
Ile	1.31	0.74 ± 0.02a	0.61 ± 0.03b	0.55 ± 0.02c	0.44 ± 0.02d	0.34 ± 0.05d	0.32 ± 0.1d
Met	0.3	0.46 ± 0.02a	0.3 ± 0.02b	0.25 ± 0.01c	0.18 ± 0.01d	0.22 ± 0.03 cd	0.19 ± 0.02de
Tyr	0.72	0.44 ± 0.01a	–	–	–	0.23 ± 0.01b	0.18 ± 0c
Phe	7.43	0.47 ± 0.01a	–	–	–	0.22 ± 0.04b	0.09 ± 0.16c
Asp	0.53	0.33 ± 0.14a	0.23 ± 0.13a	–	0.19 ± 0.07a	0.1 ± 0.17bc	–
SFAAs		19.44 ± 0.61ab	20.88 ± 0.6a	21.68 ± 2.21a	17.91 ± 0.57b	23.26 ± 0.44a	21.57 ± 2.98ab
BFAAs		8.06 ± 0.21d	23.06 ± 1.25a	16.64 ± 0.39c	18.73 ± 0.66b	18.16 ± 4b	14.71 ± 0.44c
TFAAs		27.83 ± 0.83c	44.17 ± 1.83a	38.32 ± 2.53b	36.84 ± 0.53b	41.52 ± 3.5ab	36.29 ± 3.39c

Note: Different superscript letters (a, b, c, d) indicate significant differences (P < 0.05) among different aquaculture zones. SFAAs are sweet free amino acids, BFAAs are bitter free amino acids, and TFAAs are total free amino acids. Amino acid taste thresholds are referenced from Malila et al. (2024).

Threshold values play a crucial role in taste perception by determining the extent to which amino acids influence flavor. In this study, the concentrations of Gly, Ala, His, and Ser exceeded their respective threshold values, indicating that these four amino acids had the most significant impact on fish muscle flavor. Among them, Gly—the predominant SFAAs—was most abundant in the D3L group. Conversely, His, the only bitter-tasting FAAs (BFAAs) that exceeded its threshold, had the lowest concentration in D3L. Previous research by Zhao et al. (2019) demonstrated that low-density farming reduces His levels in grass carp but may also result in the loss of Gly, Ala, and Ser. The discrepancies observed in this study could be attributed to differences in feeding restrictions and farming density selection. Compared to dietary interventions, low-density clear water depuration appears to be a more effective strategy for enhancing the concentration of SFAAs, thereby improving the overall flavor of fish muscle (Cai et al., 2024).

### 3.7 Changes in volatile components

**Fig. 4:**
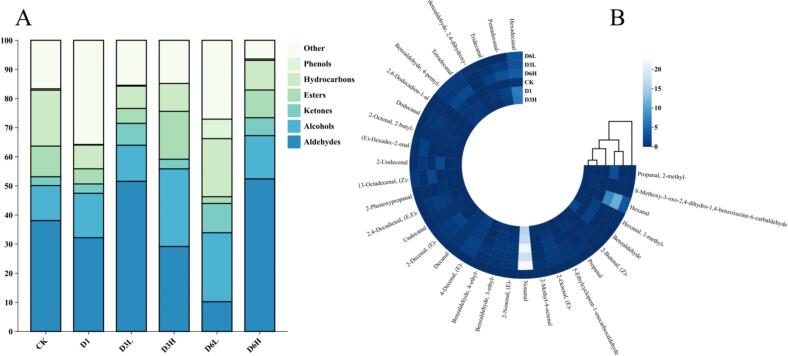
Classification of volatile components and clustering of volatile components.A shows the classification of volatile components for different groups of samples and B shows the clustering of aldehyde volatile components for different groups of samples.

A total of 335 volatile compounds were detected using HS-SPME-GC–MS. These included 30 aldehydes, 35 alcohols, 46 ketones, 48 esters, 72 hydrocarbons, 6 phenols, and 98 amines and heterocyclic compounds (Table S3). Cluster analysis revealed that aldehydes and alcohols primarily formed four distinct clusters, whereas ketones were grouped into five clusters. Notably, samples from the D3L and D6L groups clustered together, indicating a higher degree of similarity within the low-density group, which also exhibited a more pronounced difference from the CK group (Fig. 4B, Fig. S1). As the clear water depuration duration increased, the composition of volatile compounds underwent significant changes. In general, clear water depuration is employed to reduce fishy odors, which are commonly associated with compounds such as nonanal, n-hexanal, 1-octen-3-ol, (E)-2-octenal, and (E)-2-nonenal (Wu, Wang, Wang, Tian, & Zhan, 2022). After depuration, aldehyde content in D3L significantly increased compared to D3H, while alcohol content decreased. Specifically, the levels of n-hexanal and nonanal—both of which contribute to fatty and grassy aromas—were significantly higher in D3L, whereas 1-octen-3-ol, an earthy, fishy-smelling compound, was markedly reduced. After six months of farming, a distinct shift was observed. Compared to D6H, the levels of nonanal, (E)-2-octenal, and (E, E)-2,4-decadienal were undetectable in D6L, while n-hexanal and 1-octen-3-ol were significantly reduced. Relative to CK, aldehyde content in D6L decreased by 27.87 %, whereas in D6H, aldehyde levels increased by 14.31 %. Previous studies have demonstrated that starvation can effectively reduce off-flavor compounds in grass carp (Lv et al., 2018). The findings of this study suggest that prolonged clear water depuration effectively diminishes off-flavors, with low-density farming further enhancing this effect.

To further identify the characteristic volatile compounds in fish muscle, we employed the ROAV method and screened a total of 21 key volatile compounds (Table 3). In the CK group, the primary characteristic volatile compounds were (E)-2-Nonenal and (E, E)-2,4-Decadienal, both of which contribute to green and fatty aromas. After three months of clear water depuration, the characteristic volatile compounds in both D3L and D3H remained Dimethyl Trisulfide, (E)-2-Nonenal, and (E, E)-2,4-Decadienal, indicating that the fish muscle's odor profile did not change significantly during this period. However, after six months, distinct differences emerged. In D6H, (E, E)-2,4-Decadienal persisted as the dominant volatile compound, whereas D6L exhibited significant changes. The primary volatile compound in D6L was 1-Octen-3-ol, accompanied by a substantial reduction in the ROAV values of other off-flavor compounds. This shift occurred because ROAV is a relative quantification method—since the levels of other off-flavor compounds in D6L were nearly undetectable, 1-Octen-3-ol became the dominant volatile compound. In conclusion, clear water depuration effectively reduces the presence of volatile off-flavor compounds, with extended depuration periods and low-density farming conditions yielding the most pronounced improvements in odor quality.

**Table 3:** Effect of clear water depuration on ROAV of fish muscle.

Volatile Components	Threshold(mg/kg)	Odor Description	ROAV					
			CK	D1	D3L	D3H	D6L	D6H
Propanal, 2-Methyl-	0.0007	Spicy	–	–	–	–	–	15.53
Hexanal	0.0075	Pungent	–	–	9.05	–	16.61	4.11
Propanal	0.007	Pungent	–	1.51	0.37	–	–	–
(*E*)-2-Octenal	0.061	Green Leaves	0.11	0.21	0.08	–	–	0.08
Nonanal	0.035	Orange Rose	3.32	8.18	3.87	0.55	–	2.07
(E)-2-Nonenal	0.000065	Fatty	74.13	96.56	59.47	–	–	43.73
(E)-4-Decenal	0.01	Fatty	0.53	2.07	0.88	0.16	–	0.63
Decanal	0.005	Orange Peel	0.69	2.23	0.69	0.14	–	0.5
(E)-2-Decenal	0.15	Waxy Orange	0.07	–	0.03	–	0.08	0.03
Undecanal	0.014	Fatty	0.1	0.3	0.13	–	–	0.1
(E, E)-2,4-Decadienal	0.00005	Butter, Fat, Green	100	–	100	–	–	100
Dodecanal	0.001	Fatty	2.26	–	3.55	–	–	3.15
Tetradecanal	0.06	Fatty	0.02	0.21	0.09	0.02	0.77	0.14
1-Octen-3-ol	0.002	Earthy	23.22	100	21.53	6.79	100	21.95
1-Nonanol	0.002	Lemongrass Oil	0.29	–	–	–	–	0.71
2-Decanone	0.009	Peaches	0.15	0.33	0.13	–	–	0.1
5,9-Undecadien-2-One, 6,10-Dimethyl-, (E)-	0.01	Sweet Rose	–	0.3	0.05	0.03	–	–
Octanoic Acid, Methyl Ester	0.5	Wine,Orange	–	0.02	–	–	–	–
Dimethyl Trisulfide	0.000008	Spicy	–	–	–	100	–	–
Furan, 2-Pentyl-	0.0048	Fruity	4.41	–	–	–	–	–
Tetrasulfide, Dimethyl	0.0002	Garlic	3.28	11.97	5.55	2.15	–	–

### 3.8 Nutrient composition of fish in relation to sensory quality

Producers typically focus on enhancing the sensory quality of clear water fish. By implementing clear water depuration farming strategies, such as controlling farming density and adjusting farming duration, they can effectively reduce earthy off-flavors, optimize fish texture by improving hardness and chewiness, and enhance appearance by increasing the WI to meet market demands for “flavorful, odor-free” aquatic products. However, while consumers seek an improved sensory experience, they also have higher expectations for nutritional value, including protein content, EAAs retention, and a balanced fatty acid composition.

**Fig. 5:**
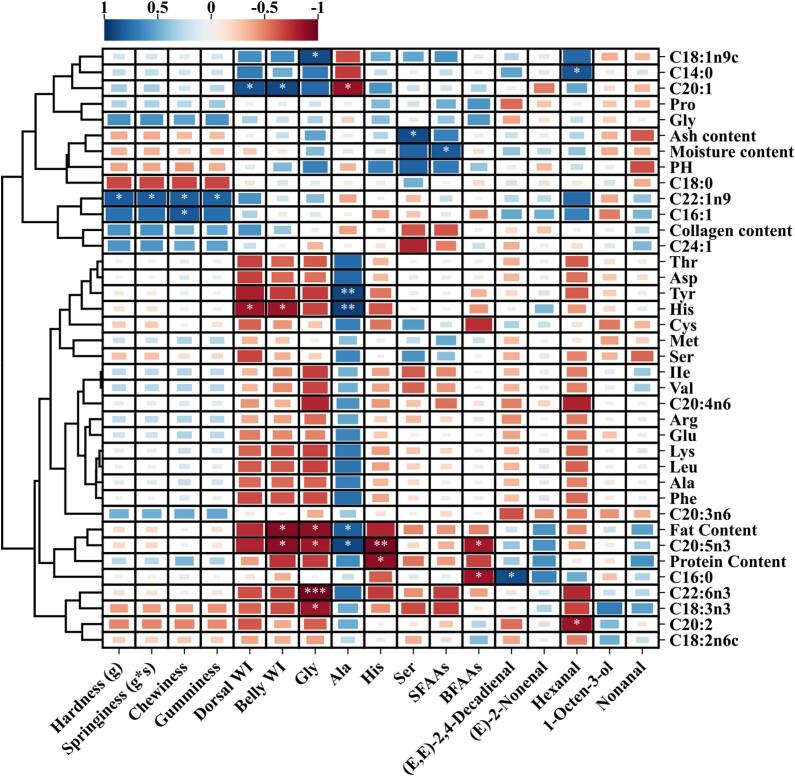
Correlation analysis between sensory quality and nutritional quality of fish muscle.

Intergroup correlation analysis revealed that improvements in fish texture were significantly positively correlated with MUFAs and protein content, while negatively correlated with fat content, moisture content, and pH (Fig. 5). These findings align with previous research. The degradation of fat reduces the intermuscular lipid droplet filling effect, promoting a denser alignment of myofibrils, which significantly enhances hardness (Fischer, Bender, Domig, & Fuhrmann, 2025; Kokoszyński et al., 2025). Higher protein content strengthens myofibrillar cross-linking and water-holding capacity, making it a key factor in increasing hardness and chewiness. Conversely, an increase in pH can weaken the water-holding ability of myofibrillar proteins, while fluctuations in moisture content, combined with freeze-thaw damage, exacerbate drip loss and negatively impact texture stability (Liu et al., 2024). Collagen content also exhibited a strong positive correlation with fish texture, suggesting that clear water depuration may promote collagen synthesis, further enhancing muscle integrity. A study by Dong et al. (2022) found that dietary protein levels in grass carp influenced collagen metabolism, thereby improving texture. However, no research has yet explored how clear water depuration directly affects collagen metabolism in grass carp. Overall, D3L effectively preserved both fish texture and flavor while minimizing nutritional losses, making it the most optimal condition.

After six months of depuration, the TFAAs content in fish muscle reached its highest level, while the TAAs content was at its lowest. Four major FAAs were strongly correlated with His, Tyr, Leu, and Lys, with His and Tyr showing the most significant relationships. These amino acids are essential for energy homeostasis, muscle protection, and overall health regulation (Nie, He, Zhang, Zhang, & Ma, 2018). The increase in FAAs may indicate that the fish were in a catabolic state, where protein breakdown exceeded synthesis, leading to greater FAAs accumulation for energy metabolism (Yu et al., 2022). This suggests that clear water depuration alters FAAs levels by inducing environmental stress, accelerating the consumption of EAAs, with prolonged depuration periods intensifying this effect.

Additionally, the study found that typical off-flavor compounds in fish muscle were significantly negatively correlated with PUFAs metabolism. Clear water depuration appears to enhance flavor by accelerating PUFAs oxidation for energy, thereby reducing the accumulation of off-flavor precursors. Research by Liu et al. (2024) on *Takifugu rubripes* demonstrated that reducing PUFAs content in fish muscle led to decreased levels of volatile lipid oxidation derivatives, such as 1-octen-3-ol, 2-hexenal, and 2-pentenal. This aligns with the current findings, indicating that volatile compound formation in fish muscle is primarily influenced by fatty acid composition. While longer depuration periods more effectively eliminate off-flavors, they may also lead to the loss of free amino acids and reduced texture quality.

## 4 Conclusion

Clear water depuration farming, as an effective method to improve the quality of freshwater fish, influences the nutritional composition of fish muscle through farming time and farming density, thereby significantly affecting the texture and flavor composition of the fish muscle. This study indicates that an extended clear water depuration time can increase the content of FAAs and substantially reduce volatile off-flavor substances. However, the loss of PUFAs and EAAs may impact the nutritional value of the product. A farming period of three months can achieve a balance between sensory quality and nutritional composition, i.e., reducing fat content and volatile off-flavor substances while maintaining relatively high levels of protein and essential amino acids, optimizing the composition of free amino acids, and improving the overall texture of the fish muscle. It is important to note that this study was conducted in Zhejiang, China, a region with a subtropical climate. Therefore, the influence of environmental temperature on fish metabolism and depuration efficiency must be considered in broader aquaculture applications. Moreover, combining clear-water depuration with low-density farming can further improve muscle quality. Based on the findings, a three-month depuration period under low-density conditions is recommended in practical farming to achieve optimal fish quality.

## CRediT authorship contribution statement

**Xinyang Li:** Writing – review & editing, Writing – original draft, Software, Formal analysis, Conceptualization. **Chenyang Zhao:** Writing – original draft, Visualization, Software, Investigation, Formal analysis, Data curation. **Lin Xu:** Investigation, Formal analysis. **Yuxiang Wang:** Visualization, Software, Investigation, Conceptualization. **Jin Yu:** Project administration, Investigation, Funding acquisition. **Xudong Weng:** Project administration, Investigation, Funding acquisition. **Ting Ye:** Investigation, Formal analysis. **Xiaoguo Ying:** Visualization, Supervision, Resources, Project administration, Methodology, Funding acquisition, Conceptualization. **Yang Gao:** Supervision, Project administration, Methodology, Data curation, Conceptualization.

## Ethical statement

This study was conducted in strict accordance with the Guidelines for Euthanasia of Laboratory Animals in the People's Republic of China (GB/T 39760-2021). In addition, the study was approved by the Ethics Committee for Experimental Animals of Zhejiang Ocean University (Certificate No. SCXK Z HE2014-0001).

## Declaration of competing interest

The authors declare that they have no known competing financial interests or personal relationships that could have appeared to influence the work reported in this paper.

## Data Availability

Data will be made available on request.

## Appendix A Supplementary data

Supplementary material Image 1
