# Supplementary material for: A method for improving the quality of grass carp (Ctenopharyngodon idellus): A comprehensive evaluation of clear water depuration based on sensory and nutritional aspects
Source: Food Chem X. 2025 May 29;28:102601. doi: 10.1016/j.fochx.2025.102601 (PMC12163169; doi:10.1016/j.fochx.2025.102601)
Supplement: Supplementary file 1 — Supplementary material [file mmc1.docx]

**Title:** A method for improving the quality of grass carp (*Ctenopharyngodon idellus*): a comprehensive evaluation of clear water depuration based on sensory and nutritional aspects

**Author names:**

Xinyang Li^a, 1^, Chenyang Zhao^a, 1^, Lin Xu^a^, Yuxiang Wang^a^, Jin Yu^c^, Xudong Weng^d, *^, Ting Ye^e^, Xiaoguo Ying^a, *^, Yang Gao^b, *^

**Author affiliations:**

^a^ Zhejiang Provincial Key Laboratory of Health Risk Factors for Seafood, Collaborative Innovation Center of Seafood Deep Processing, College of Food and Pharmacy, Zhejiang Ocean University, Zhoushan, China

^b^ College of Fishery, Zhejiang Ocean University, Zhoushan, China

^c^ Longyou Aquaculture Development Center, Agricultural and Rural Bureau of Longyou County, Quzhou, China

^d^ Zhejiang Yulaoda Agricultural Technology Co., Ltd., Quzhou, China

^e^ Quzhou Aquatic Technology Extension Centre, Quzhou, China

^1^ These authors contributed equally to this work.

**Contact information for the Corresponding author:**

Dr. Xiaoguo Ying

Email address: yingxiaoguo@zjou.edu.cn

Postal address: No.1, Haida South Road, Lincheng Changzhi Island, Zhoushan, Zhejiang province, 316022 P. R. China.

Dr. Yang Gao

Email address: gaoyang82@zjou.edu.cn

Postal address: No.1, Haida South Road, Lincheng Changzhi Island, Zhoushan, Zhejiang province, 316022 P. R. China.

Dr. Xudong Weng

Email address: 707544643@qq.com

Postal address: No.319, Longlan Road, Huzhen Town, Longyou County, Quzhou, Zhejiang Province, 324400 P. R. China.

Figure S1 Clustering of volatile components of different groups of samples. A is alcohols, B is ketones.


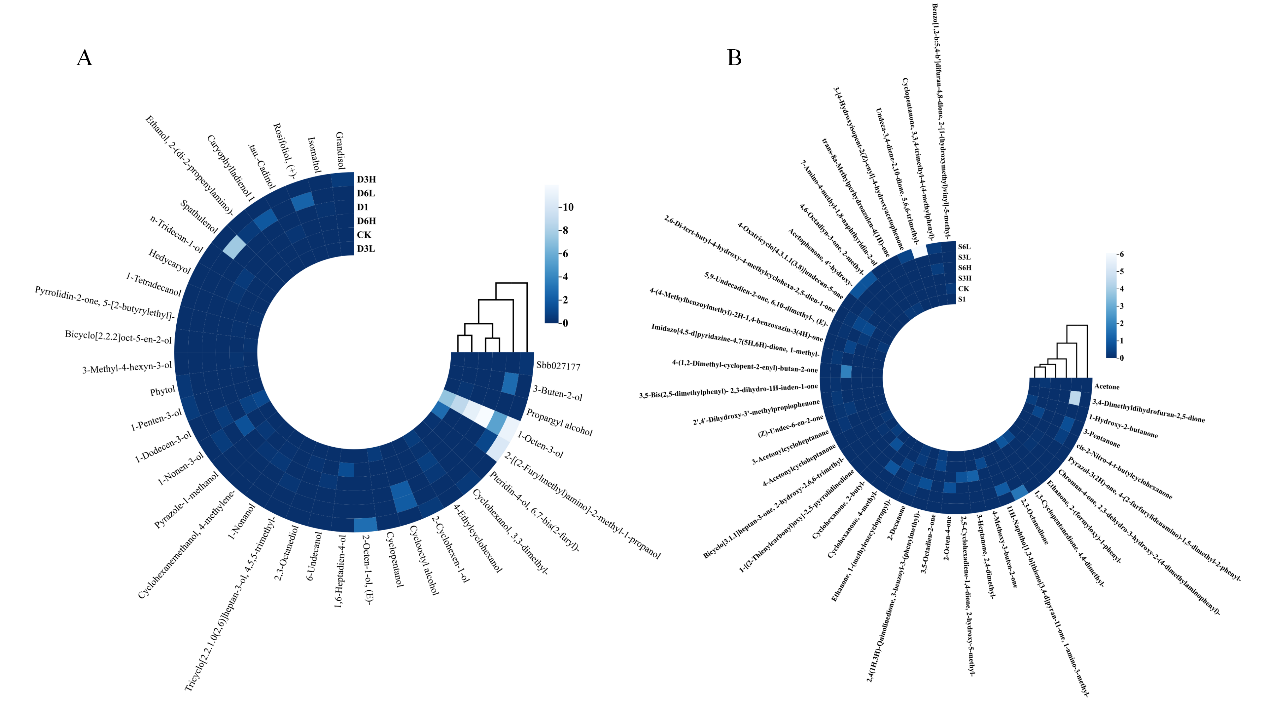


Table S1 Growth characteristics of fish after clear water depuration.

| Growth characteristics | CK | D1 | D3L | D3H | D6L | D6H |
| --- | --- | --- | --- | --- | --- | --- |
| Body weight/g | 1059.65±37.26a | 882.8±13.03b | 992.67±38.15a | 912.82±60.4a | 904.85±257.98a | 1046.28±185.57a |
| Visceral weight/g | 92.18±10.21a | 49.75±0.57b | 53.57±8.24b | 49.73±7.13b | 61.91±14.8b | 60.95±15.5b |
| Body length/cm | 45.17±2.47ab | 43.9±1.68ab | 46.47±0.12a | 44.4±1.31ab | 39.87±7.27b | 43.07±1.62ab |
| Body width/cm | 9.17±0.29a | 8.37±0.65ab | 8.43±0.61ab | 7.57±0.42b | 8.27±0.4b | 8.2±0.1b |
| Body thickness/cm | 5.77±0.25bc | 5.73±0.75bc | 6.47±1.02ab | 6.9±0.1a | 5.33±0.38c | 5±0.36c |
| Condition factor | 1.16±0.15a | 1.05±0.13a | 0.99±0.04a | 1.05±0.15a | 1.48±0.43a | 1.34±0.4a |
| Viscerosomatic index | 0.09±0.01a | 0.06±0b | 0.05±0.01b | 0.05±0b | 0.07±0.02ab | 0.06±0.01b |

Note：Different superscript letters (a, b) indicate significant differences (P < 0.05) among different aquaculture zones.

Table S2 Nutritional composition of fish muscle

| Nutritional compositions | CK | D1 | D3L | D3H | D6L | D6H |
| --- | --- | --- | --- | --- | --- | --- |
| PH | 6.35±0.1a | 6.77±0.03b | 6.52±0.06c | 6.52±0.16c | 7.05±0.08a | 7.06±0.03a |
| Moisture content | 76.83±0.62a | 76.83±0.62a | 76.5±3.42a | 72.17±4.36b | 80.09±0.47a | 80.72±0.45a |
| Crude fat content | 2.35±0.2a | 1.52±0.08b | 0.94±0.08c | 0.82±0.04c | 0.45±0.07d | 0.62±0.03d |
| Ash content | 0.98±0.06b | 0.85±0.03cd | 0.89±0.04c | 0.81±0.02d | 2.15±0.02a | 2.18±0.05a |
| Crude protein content (%) | 19.65±0.2a | 16.31±0.18c | 17.89±0.25b | 16.58±0.27c | 15.56±0.02d | 15.49±0.02d |
| Collagen content （g/kg） | 4.41±0.002c | 3.61±0.002e | 6.05±0.002b | 7.38±0.003a | 3.75±0.006d | 3.5±0.004f |

Note：Different superscript letters (a, b, c, d, e) indicate significant differences (P < 0.05) among different aquaculture zones.

Table S3 Volatile components of different groups of samples.

| Volatile components | CAS number | RI | Relative content | | | | | |
| --- | --- | --- | --- | --- | --- | --- | --- | --- |
|  |  |  | CK | D1 | D3L | D3H | D6L | D6H |
| **Aldehydes** |  |  |  |  |  |  |  |  |
| Propanal, 2-methyl- | 78-84-2 | 4.171 | - | - | - | - | - | 2.696 |
| 8-Methoxy-3-oxo-2,4-dihydro-1,4-benzoxazine-6-carbaldehyde | 711021-34-0 | 4.331 | - | 0.096 | - | - | - | - |
| Hexanal | 66-25-1 | 4.44 | - | - | 11.307 | - | 3.328 | 7.653 |
| Hexanal, 3-methyl- | 19269-28-4 | 6.569 | 1.384 | - | - | - | - | - |
| Benzaldehyde | 100-52-7 | 8.123 | 1.643 | - | - | 1.917 | - | - |
| 2-Butenal, (Z)- | 15798-64-8 | 8.152 | - | - | - | - | - | 0.278 |
| Propanal | 123-38-6 | 8.962 | - | 0.63 | 0.436 | - | - | - |
| 5-Ethylcyclopent-1-enecarboxaldehyde | 36431-60-4 | 10.111 | - | - | 0.513 | - | - | 0.515 |
| 2-Octenal, (E)- | 2548-87-0 | 10.845 | 1.287 | 0.759 | 0.812 | - | - | 1.2 |
| 2-Methyl-4-octenal | 30390-58-0 | 11.92 | - | - | 0.397 | - | - | - |
| Nonanal | 124-19-6 | 12.177 | 21.831 | 17.097 | 22.578 | 15.867 | - | 17.958 |
| 2-Nonenal, (E)- | 18829-56-6 | 13.773 | 0.904 | 0.375 | 0.644 | - | - | 0.705 |
| Benzaldehyde, 3-ethyl- | 34246-54-3 | 13.892 | 0.845 | 0.565 | 0.455 | 0.581 | - | 0.726 |
| Benzaldehyde, 4-ethyl- | 4748-78-1 | 13.903 | - | - | 0.409 | - | - | 0.707 |
| 4-Decenal, (E)- | 65405-70-1 | 14.755 | 1.002 | 1.239 | 1.461 | 1.332 | - | 1.552 |
| Decanal | 112-31-2 | 15.083 | 0.648 | 0.665 | 0.573 | 0.565 | - | 0.615 |
| 2-Decenal, (E)- | 3913-81-3 | 16.642 | 1.831 | - | 0.873 | - | 0.324 | 1.052 |
| Undecanal | 112-44-7 | 17.88 | 0.27 | 0.251 | 0.307 | - | - | 0.362 |
| 2,4-Decadienal, (E,E)- | 25152-84-5 | 18.143 | 0.938 | - | 0.833 | - | - | 1.24 |
| 2-Phenoxypropanal | 52687-81-7 | 18.185 | 0.398 | - | 0.173 | - | - | - |
| 13-Octadecenal, (Z)- | 58594-45-9 | 19.158 | - | - | - | - | 0.857 | 2.083 |
| 2-Undecenal | 2463-77-6 | 19.382 | 2.299 | - | 0.873 | - | - | - |
| (E)-Hexadec-2-enal | 22644-96-8 | 19.384 | - | - | 1.588 | - | - | - |
| 2-Octenal, 2-butyl- | 13019-16-4 | 19.653 | 0.283 | 0.377 | 0.19 | - | - | 0.156 |
| Dodecanal | 112-54-9 | 20.541 | 0.424 | - | 0.592 | - | - | 0.78 |
| 2,6-Dodecadien-1-al | 21662-13-5 | 21.526 | - | - | 0.112 | - | - | - |
| Benzaldehyde, 4-pentyl- | 6853-57-2 | 21.871 | 0.849 | 0.567 | 0.802 | 0.987 | 0.997 | 1.815 |
| Tetradecanal | 124-25-4 | 25.455 | 0.27 | 0.764 | 0.889 | 0.773 | 1.234 | 2.111 |
| Benzaldehyde, 2,4-dihydroxy- | 95-01-2 | 24.676 | - | - | - | - | 0.404 | - |
| Tridecanal | 10486-19-8 | 23.06 | 0.231 | 0.572 | 0.555 | - | - | 1.397 |
| Pentadecanal- | 2765-11-9 | 27.735 | 0.255 | 1.575 | 1.232 | 1.065 | - | 2.893 |
| Hexadecanal | 629-80-1 | 30.181 | 0.492 | 6.668 | 2.972 | 6.095 | 3.072 | 3.902 |
| **Alcohols** |  |  | - | - | - | - | - | - |
| [3-(4-Methoxyphenyl)-4,5-dihydro-1,2-oxazol-5-yl]methanol | 206055-84-7 | 3.564 | - | - | - | 0.242 | - | - |
| 3-Buten-2-ol | 598-32-3 | 4.236 | - | - | - | - | 2.684 | - |
| Propargyl alcohol | 107-19-7 | 4.522 | 0.227 | - | - | - | - | - |
| 1-Octen-3-ol | 3391-86-4 | 8.623 | 8.713 | 11.949 | 7.173 | 11.241 | 5.343 | 10.886 |
| 2-[(2-Furylmethyl)amino]-2-methyl-1-propanol | 889949-94-4 | 8.945 | - | - | 2.736 | 10.211 | 0.978 | - |
| Pteridin-4-ol, 6,7-bis(2-furyl)- | 313996-29-1 | 8.949 | - | - | 0.151 | - | - | - |
| Cyclohexanol, 3,3-dimethyl- | 767-12-4 | 10.138 | - | - | - | 0.4 | - | - |
| 4-Ethylcyclohexanol | 4534-74-1 | 10.432 | 0.136 | 0.372 | 0.538 | - | - | 0.661 |
| 2-Cyclohexen-1-ol | 822-67-3 | 10.884 | - | - | - | 0.437 | - | - |
| Cyclooctyl alcohol | 696-71-9 | 11.148 | - | 2.028 | - | - | 2.01 | - |
| Cyclopentanol | 96-41-3 | 11.155 | - | - | 0.455 | - | - | - |
| 2-Octen-1-ol, (E)- | 18409-17-1 | 11.193 | - | - | - | 2.838 | - | - |
| 1,6-Heptadien-4-ol | 2883-45-6 | 11.199 | 1.258 | - | - | - | - | - |
| 6-Undecanol | 23708-56-7 | 11.706 | - | - | - | - | - | 0.337 |
| 2,3-Octanediol | 20653-90-1 | 11.901 | 0.2 | - | - | - | - | - |
| Tricyclo[2.2.1.0(2,6)]heptan-3-ol, 4,5,5-trimethyl- | 62560-53-6 | 13.365 | - | 0.264 | - | - | - | - |
| 1-Nonanol | 143-08-8 | 14.115 | 0.107 | - | - | - | - | 0.353 |
| Cyclohexanemethanol, 4-methylene- | 1004-24-6 | 14.616 | - | - | - | - | - | 0.239 |
| Pyrazole-1-methanol | 1120-82-7 | 16.645 | 0.383 | - | - | - | - | - |
| 1-Nonen-3-ol | 21964-44-3 | 16.964 | - | - | - | - | 0.535 | 1.48 |
| 1-Dodecen-3-ol | 4048-42-4 | 17.174 | 0.763 | 0.532 | 1.11 | - | - | - |
| 1-Penten-3-ol | 616-25-1 | 17.2 | - | - | - | 0.485 | - | - |
| Phytol | 150-86-7 | 17.878 | - | - | - | 0.336 | - | - |
| 3-Methyl-4-hexyn-3-ol | 6320-68-9 | 18.86 | 0.167 | - | - | - | - | - |
| Bicyclo[2.2.2]oct-5-en-2-ol | 55320-40-6 | 19.332 | - | - | 0.126 | - | - | - |
| Pyrrolidin-2-one, 5-[2-butyrylethyl]- | 117155-75-6 | 25.145 | 0.079 | - | - | - | - | - |
| 1-Tetradecanol | 112-72-1 | 22.602 | - | - | 0.114 | - | - | - |
| 3,7-Cyclodecadiene-1-methanol, .alpha.,.alpha.,4,8-tetramethyl-, [s-(Z,Z)] | 21657-90-9 | 23.845 | - | - | - | - | - | 0.22 |
| n-Tridecan-1-ol | 112-70-9 | 24.366 | - | - | - | - | - | 0.473 |
| 1H-Cycloprop[e]azulen-7-ol, decahydro-1,1,7-trimethyl-4-methylene-, [1ar-(1a.alpha.,4a.alpha.,7.beta.,7a.beta.,7b.alpha.)]- | 6750-60-3 | 24.56 | - | - | - | - | 7.546 | - |
| Ethanol, 2-(di-2-propenylamino)- | 17719-79-8 | 25.227 | - | - | - | - | 0.457 | - |
| 10,10-Dimethyl-2,6-dimethylenebicyclo[7.2.0]undecan-5.beta.-ol | 19431-80-2 | 25.918 | - | - | - | - | 1.804 | - |
| .tau.-Cadinol | 5937-11-1 | 25.993 | - | - | - | - | - | 0.231 |
| 2-Naphthalenemethanol, 2,3,4,4a,5,6,7,8-octahydro-.alpha.,.alpha.,4a,8-tetramethyl-, [2R-(2.alpha.,4a.beta.,8.beta.)]- | 63891-61-2 | 26.229 | - | - | - | - | 2.331 | - |
| Isomaltol | 3420-59-5 | 27.381 | - | 0.117 | - | - | - | - |
| Grandisol | 26532-22-9 | 27.722 | - | - | - | 0.478 | - | - |
| **Ketones** |  |  | - | - | - | - | - | - |
| Acetone | 67-64-1 | 4.339 | 0.132 | - | - | - | - | - |
| 3,4-Dimethyldihydrofuran-2,5-dione | 7475-92-5 | 4.391 | - | - | 4.41 | - | - | - |
| 1-Hydroxy-2-butanone | 5077-67-8 | 4.406 | - | 0.315 | - | - | - | - |
| 3-Pentanone | 96-22-0 | 6.607 | 0.507 | - | 0.695 | - | - | - |
| cis-2-Nitro-4-t-butylcyclohexanone | 74609-73-7 | 6.622 | - | - | 0.342 | - | - | - |
| Pyrazol-3(2H)-one, 4-(2-furfurylidenamino)-1,5-dimethyl-2-phenyl- | 92968-42-8 | 7.028 | - | - | - | 0.064 | - | - |
| Chroman-4-one, 2,3-dehydro-3-hydroxy-2-(4-dimethylaminophenyl)- | 101442-35-7 | 7.238 | - | - | - | 0.109 | - | - |
| Ethanone, 2-(formyloxy)-1-phenyl- | 55153-12-3 | 8.110 | - | 0.533 | - | - | - | - |
| 1,3-Cyclopentanedione, 4,4-dimethyl- | 4683-51-6 | 8.355 | - | 0.872 | - | - | - | - |
| 2,3-Octanedione | 585-25-1 | 8.461 | - | - | - | - | 1.627 | - |
| 11H-Naphtho[1,2-b]thieno[3,4-d]pyran-11-one, 1-amino-3-methyl- | 292159-63-8 | 8.581 | - | - | 0.998 | - | - | - |
| 4-Methoxy-3-buten-2-one | 4652-27-1 | 8.615 | 0.579 | - | - | - | - | - |
| 3-Heptanone, 2,4-dimethyl- | 18641-71-9 | 8.773 | - | - | - | 1.168 | - | - |
| 2,5-Cyclohexadiene-1,4-dione, 2-hydroxy-5-methyl- | 615-91-8 | 8.948 | - | - | - | 0.809 | - | - |
| 2-Octen-4-one | 4643-27-0 | 10.997 | - | - | - | - | - | 0.75 |
| 3,5-Octadien-2-one | 38284-27-4 | 11.944 | 0.491 | 0.308 | - | - | - | - |
| 2,4(1H,3H)-Quinolinedione, 3-benzoyl-3-(phenylmethyl)- | 70611-42-6 | 13.406 | - | - | - | - | - | 0.707 |
| 2-Decanone | 693-54-9 | 14.72 | 0.259 | 0.18 | 0.194 | - | - | 0.228 |
| Ethanone, 1-(methylenecyclopropyl)- | 62266-35-7 | 14.779 | - | - | - | 0.361 | - | - |
| Cyclohexanone, 4-methyl- | 589-92-4 | 16.552 | - | - | - | - | - | 0.858 |
| Cyclohexanone, 2-butyl- | 1126-18-7 | 16.766 | 0.739 | - | - | - | - | - |
| 1-[(2-Thienylcarbonyl)oxy]-2,5-pyrrolidinedione | 83039-60-5 | 16.769 | - | 0.266 | - | - | - | - |
| Bicyclo[3.1.1]heptan-3-one, 2-hydroxy-2,6,6-trimethyl- | 10136-65-9 | 16.925 | 0.096 | - | - | - | - | - |
| 4-Acetonylcycloheptanone | 86428-60-6 | 17.069 | - | 0.161 | - | - | - | - |
| 3-Acetonylcycloheptanone | 66921-76-4 | 17.077 | - | - | 0.148 | - | - | - |
| (Z)-Undec-6-en-2-one | 107853-70-3 | 17.086 | - | - | 0.105 | - | - | 0.204 |
| 2',4'-Dihydroxy-3'-methylpropiophenone | 63876-46-0 | 17.522 | - | - | 0.175 | - | - | - |
| 3,5-Bis(2,5-dimethylphenyl)- 2,3-dihydro-1H-inden-1-one | 357941-13-0 | 18.42 | - | - | 0.064 | - | - | - |
| 4-(1,2-Dimethyl-cyclopent-2-enyl)-butan-2-one | 75698-06-5 | 20.105 | - | - | - | - | - | 1.887 |
| Imidazo[4,5-d]pyridazine-4,7(5H,6H)-dione, 1-methyl- | 6286-08-4 | 20.338 | - | - | 0.178 | - | - | - |
| 4-(4-Methylbenzoylmethyl)-2H-1,4-benzoxazin-3(4H)-one | 105492-44-2 | 21.632 | - | - | - | - | - | 0.462 |
| 5,9-Undecadien-2-one, 6,10-dimethyl-, (E)- | 3796-70-1 | 21.655 | - | 0.182 | 0.086 | 0.208 | - | - |
| 2,6-Di-tert-butyl-4-hydroxy-4-methylcyclohexa-2,5-dien-1-one | 10396-80-2 | 22.164 | - | - | 0.108 | 0.592 | - | 0.53 |
| 4-Oxatricyclo[4.3.1.1(3,8)]undecan-5-one | 21898-84-0 | 22.236 | - | - | - | - | - | 0.207 |
| Acetophenone, 4'-hydroxy- | 99-93-4 | 22.585 | - | - | - | - | 0.825 | - |
| 4,6-Octadiyn-3-one, 2-methyl- | 29743-33-7 | 24.681 | - | - | - | - | 0.827 | - |
| 7-Amino-4-methyl-1,8-naphthyridin-2-ol | 1569-15-9 | 21.870 | - | 0.2 | - | - | - | - |
| trans-8a-Methylperhydroazulen-4(1H)-one | 32166-45-3 | 25.450 | - | 0.191 | - | - | - | - |
| 3-[4-Hydroxyisopent-2(Z)-enyl]-4-hydroxyacetophenone | 24672-83-1 | 25.929 | - | - | - | - | 0.461 | - |
| Undeca-3,4-diene-2,10-dione, 5,6,6-trimethyl- | 90165-10-9 | 26.022 | - | - | - | - | 6.064 | - |
| Cyclopentanone, 3,3,4-trimethyl-4-(4-methylphenyl)- | 56077-23-7 | 28.781 | - | - | - | - | 0.262 | 0.3 |
| Benzo[1,2-b:5,4-b']difuran-4,8-dione, 2-[1-(hydroxymethyl)vinyl]-5-methyl- | 26962-41-4 | 36.044 | 0.217 | - | - | - | - | - |
| **Esters** |  |  | - | - | - | - | - | - |
| 3-Hexen-1-ol, propanoate, (Z)- | 33467-74-2 | 4.314 | - | - | - | 1.335 | - | - |
| 1,4-Dioxane-2,5-dione, 3,6-dimethyl-, (3S-cis)- | 4511-42-6 | 4.368 | 3.262 | - | - | - | - | - |
| Hydrogen isocyanate | 75-13-8 | 4.431 | 0.808 | - | - | 1.656 | - | - |
| DL-Norvaline, ethyl ester | 13893-43-1 | 4.445 | 0.641 | 0.497 | - | - | - | - |
| 2-Propenoic acid, 2-methoxyethyl ester | 3121-61-7 | 4.560 | - | 0.404 | - | - | - | - |
| Methyl 2-butynoate | 23326-27-4 | 4.571 | - | - | 0.308 | - | - | - |
| 9,10-Dihydrophenanthren-2-butyric acid, methyl ester | 35639-15-7 | 7.176 | - | - | - | 0.061 | - | - |
| p-Hexyloxyphenyl trans-4-butylcyclohexanecarboxylate | 67589-50-8 | 7.77 | - | - | - | - | - | 0.271 |
| 1,3-Benzenediol, monobenzoate | 136-36-7 | 7.892 | - | - | - | - | - | 0.936 |
| Vinyl benzoate | 769-78-8 | 8.112 | - | 0.528 | - | - | - | - |
| 2-Vinylethyl acetate | 1576-84-7 | 8.482 | - | - | - | - | - | 1.607 |
| 2-Propenoic acid, oxiranylmethyl ester | 106-90-1 | 8.571 | - | - | - | 1.535 | - | - |
| n-Propyl acrylate | 925-60-0 | 8.632 | 0.691 | - | - | - | - | - |
| 2H-Pyran-2,3-diol, tetrahydro-, diacetate, cis- | 2396-74-9 | 8.761 | 0.201 | - | - | - | - | - |
| Butyric acid, ester with p-hydroxybenzonitrile | 29052-10-6 | 8.775 | - | - | 0.343 | - | - | - |
| 2-Butenedioic acid (Z)-, dimethyl ester | 624-48-6 | 8.952 | - | - | - | 1.424 | - | - |
| Geranyl tiglate | 7785-33-3 | 10.003 | 0.277 | - | - | - | - | - |
| Sulfurous acid, dimethyl ester | 616-42-2 | 11.139 | - | 0.239 | - | - | - | - |
| Formic acid, octyl ester | 112-32-3 | 11.205 | 2.996 | - | 2.181 | - | - | 3.458 |
| Octanoic acid, methyl ester | 111-11-5 | 12.783 | 0.413 | 0.477 | 0.147 | - | - | - |
| 2-Furancarboxylic acid, butyl ester | 583-33-5 | 15.078 | 0.186 | - | - | - | - | - |
| 11,14,17-Eicosatrienoic acid, methyl ester | 55682-88-7 | 15.619 | 0.237 | - | - | - | - | - |
| Nonanoic acid, methyl ester | 1731-84-6 | 15.625 | - | - | - | 0.534 | - | - |
| 2-Propenoic acid, 6-methylheptyl ester | 54774-91-3 | 15.764 | 0.118 | - | - | - | - | - |
| 2-Propyn-1-ol, propionate | 1932-92-9 | 16.776 | 0.264 | - | - | 0.76 | - | - |
| Hexanoic acid, pentyl ester | 540-07-8 | 17.361 | - | - | 0.117 | - | - | - |
| Phenol, 2-methoxy-4-(2-propenyl)-, acetate | 93-28-7 | 19.276 | 0.145 | - | - | - | - | - |
| 3-(4-Methylbenzoyl)-2-thioxo-4-thiazolyl 4-methylbenzoate | 299929-13-8 | 21.883 | - | 0.195 | - | - | - | - |
| Methyl p-(2-phenyl-1-benzimidazolyl)benzoate | 3510-24-5 | 22.543 | - | - | - | - | 1.014 | - |
| Sulfuric acid, dimethyl ester | 77-78-1 | 12.168 | - | - | - | 5.426 | - | - |
| 2,2,4-Trimethyl-1,3-pentanediol diisobutyrate | 6846-50-0 | 25.151 | - | - | - | 0.198 | - | - |
| Ethanethioic acid, S-(10-cyanodecyl) ester | 125611-10-1 | 26.763 | - | - | - | - | - | 0.553 |
| Methyl tetradecanoate | 124-10-7 | 27.952 | 0.125 | 0.318 | 0.258 | 0.63 | - | 0.424 |
| Hexyl methyl methylphosphonate | 133736-02-4 | 29.876 | - | - | - | - | 0.644 | - |
| 9-Hexadecenoic acid, methyl ester, (Z)- | 1120-25-8 | 33.009 | - | - | 0.279 | - | - | - |
| 11-Hexadecenoic acid, methyl ester | 55000-42-5 | 33.021 | - | - | - | 0.529 | - | - |
| Hexadecanoic acid, methyl ester | 112-39-0 | 33.736 | 0.172 | 1.591 | 0.982 | 1.604 | 0.651 | 1.48 |
| 6-Octadecenoic acid, methyl ester, (Z)- | 2777-58-4 | 37.206 | - | - | - | - | - | 0.375 |
| 9,11-Octadecadienoic acid, methyl ester, (E,E)- | 13038-47-6 | 37.299 | - | 0.413 | - | - | - | - |
| 9,12-Octadecadienoic acid (Z,Z)-, methyl ester | 112-63-0 | 37.301 | - | - | 0.222 | - | - | 0.422 |
| 9,12-Octadecadienoic acid, methyl ester | 2462-85-3 | 37.312 | - | - | - | 0.285 | - | - |
| 11-Octadecenoic acid, methyl ester | 52380-33-3 | 37.394 | - | - | 0.27 | - | - | - |
| 13-Octadecenoic acid, methyl ester | 56554-47-3 | 37.396 | - | - | - | 0.461 | - | - |
| 9-Octadecenoic acid, methyl ester, (E)- | 1937-62-8 | 37.398 | - | 0.536 | - | - | - | - |
| **Hydrocarbons** |  |  | - | - | - | - | - | - |
| Oxazolidine, 2-methyl- | 16250-70-7 | 4.072 | - | - | - | - | - | 1.171 |
| Octodrine | 543-82-8 | 4.215 | - | - | - | - | - | 1.316 |
| Propane, 1,3-dimethoxy- | 17081-21-9 | 4.374 | 0.273 | - | - | - | - | - |
| Diazene, bis(1,1-dimethylethyl)- | 927-83-3 | 4.398 | 12.652 | - | - | - | - | - |
| 2-Butanamine, 3,3-dimethyl- | 3850-30-4 | 6.516 | - | 0.294 | - | - | - | - |
| N-Methyl-2-isopropoxycarbonylazetidine | 51764-30-8 | 6.523 | - | - | 0.356 | - | - | - |
| Cyclobutane, 1,1,2,3,3-pentamethyl- | 57905-86-9 | 8.189 | - | - | - | - | - | 0.42 |
| 1-Heptene, 2,6-dimethyl- | 3074-78-0 | 8.364 | 0.75 | - | - | - | - | - |
| 3-Amino-2,4-dimethylpentane | 4083-57-2 | 8.398 | - | - | 1.967 | - | - | 1.985 |
| 3,4-Diacetylfurazan | 6102-98-3 | 8.921 | - | - | - | 2.508 | - | - |
| Oxirane, methyl-, (S)- | 16088-62-3 | 8.947 | 0.747 | - | - | - | - | - |
| 3-Methoxy-1-pentene | 14092-18-3 | 8.966 | - | - | - | 1.766 | - | - |
| Cyclohexene, 4-methyl-1-(1-methylethenyl)- | 586-67-4 | 10.02 | - | - | 0.294 | - | - | - |
| 1,3-Hexadiene, 3-ethyl-2-methyl- | 61142-36-7 | 10.089 | - | 0.67 | - | - | - | - |
| 6-Azabicyclo[3.2.1]octane | 279-85-6 | 10.835 | - | - | 0.222 | - | - | - |
| Pentadecane, 7-methyl- | 6165-40-8 | 11.713 | 0.219 | - | - | 0.283 | - | - |
| Octane, 5-ethyl-2-methyl- | 62016-18-6 | 12.017 | 0.161 | - | 0.076 | - | - | - |
| Undecane, 4,7-dimethyl- | 17301-32-5 | 12.814 | - | - | - | 0.96 | - | - |
| 1-butoxycyclohexene | 24159-57-7 | 13.808 | - | - | - | 0.413 | - | - |
| Tridecane, 2,5-dimethyl- | 56292-66-1 | 14.062 | 0.33 | - | - | - | - | - |
| Cyclopropane, 1-methyl-2-(1-methylpentyl)- | 62238-06-6 | 14.122 | - | - | - | 0.252 | - | - |
| Cycloheptane, methyl- | 4126-78-7 | 14.144 | - | - | 0.173 | - | - | - |
| (3E,5Z)-1,3,5-Undecatriene | 51447-08-6 | 14.448 | - | - | 0.073 | - | - | - |
| 1,3-Cyclohexadiene, 5-butyl- | 30168-57-1 | 14.453 | - | 0.135 | - | - | - | - |
| Bicyclo(3.3.1)non-2-ene | 6671-66-5 | 14.454 | - | - | 0.078 | - | - | - |
| 1-(2-Propenyl)cyclopentene | 37689-19-3 | 14.615 | - | - | - | - | - | 0.312 |
| Bicyclo[4.1.0]heptane, 7-methylene- | 54211-14-2 | 14.842 | 0.169 | - | - | - | - | 0.635 |
| Cyclohexene, 3,4-diethenyl-1,6-dimethyl- | 61142-14-1 | 14.854 | - | 0.17 | - | - | - | - |
| di-t-Butylacetylene | 17530-24-4 | 14.886 | - | - | - | 0.304 | - | - |
| Dodecane | 112-40-3 | 14.895 | 0.292 | 0.259 | 0.236 | - | - | 0.247 |
| 3-Heptene, 4-methyl- | 4485-16-9 | 15.082 | - | - | 0.217 | - | - | - |
| 1,2-Pentadiene, 4-methoxy-4-methyl- | 49833-91-2 | 16.651 | - | 0.172 | - | - | - | - |
| Cyclopropane, 1-butyl-1-methyl-2-propyl- | 41977-34-8 | 16.772 | - | 0.681 | 0.522 | - | 0.52 | - |
| Undecane, 3,5-dimethyl- | 17312-81-1 | 17.414 | - | - | - | - | - | 0.251 |
| Tridecane | 629-50-5 | 17.651 | 0.278 | - | 0.192 | 0.687 | - | - |
| Cyclopropene, 3,3-diethyl- | 78578-86-6 | 17.911 | - | - | - | - | - | 0.345 |
| Butane, 1,4-bis(dicyclopentylphosphino)- | 163106-88-5 | 18.176 | - | - | - | - | - | 0.324 |
| Bicyclo[2.2.2]octane, 1-methoxy-4-methyl- | 6555-95-9 | 18.871 | - | - | 0.361 | - | - | 0.846 |
| (3Z,5E)-1,3,5-Undecatriene | 19883-27-3 | 19.330 | - | 0.366 | 0.184 | - | - | - |
| (E,E)-1,3,5-Undecatriene | 19883-29-5 | 19.344 | - | - | - | 0.397 | 0.488 | - |
| Bicyclo[2.2.1]heptane, 2-(1,1-dimethyl-2-propenyl)- | 69219-08-5 | 19.386 | - | - | - | 0.272 | - | - |
| 1-Octadecyne | 629-89-0 | 19.394 | - | 0.922 | - | - | - | - |
| Tridecane, 3-methyl- | 6418-41-3 | 19.52 | 0.14 | - | - | - | - | - |
| Decane, 3,8-dimethyl- | 17312-55-9 | 20.278 | - | 0.154 | - | - | - | - |
| Tetradecane | 629-59-4 | 20.28 | 0.184 | - | 0.174 | 0.274 | - | 0.387 |
| Cyclopentene, 5-hexyl-3,3-dimethyl- | 61142-66-3 | 20.334 | 0.958 | - | 0.958 | - | 0.928 | - |
| Bicyclo[2.2.1]heptane, 2-(1-buten-3-yl)- | 55170-90-6 | 20.339 | - | 1.085 | - | - | - | - |
| Dodecane, 2-cyclohexyl- | 13151-82-1 | 20.544 | - | - | - | 0.335 | - | - |
| Bicyclo[5.2.0]nonane, 2-methylene-4,8,8-trimethyl-4-vinyl- | 242794-76-9 | 20.668 | - | - | - | - | 15.686 | - |
| 2-Epi-.alpha.-funebrene | 65354-33-8 | 20.743 | 0.078 | - | 0.092 | - | - | - |
| (1R,2S,6S,7S,8S)-8-Isopropyl-1-methyl-3-methylenetricyclo[4.4.0.02,7]decane-rel- | 18252-44-3 | 20.899 | - | - | - | - | 0.796 | - |
| Cyclohexane, 1-ethyl-1,4-dimethyl-, cis- | 62238-30-6 | 21.287 | - | - | - | - | - | 0.268 |
| Cyclohexane, (1,2-dimethylpropyl)- | 51284-29-8 | 21.522 | 0.126 | - | - | - | - | - |
| Nonane, 2,2,4,4,6,8,8-heptamethyl- | 4390-04-9 | 22.766 | - | - | - | 0.218 | - | - |
| Undecane, 5,5-dimethyl- | 17312-73-1 | 22.771 | - | - | 0.197 | - | - | - |
| Pentadecane | 629-62-9 | 22.775 | 0.352 | - | - | - | - | - |
| Nonane, 2,2,4,4,6,8,8-heptamethyl- | 4390-04-9 | 22.786 | - | 0.193 | - | - | - | - |
| Oxirane, hexadecyl- | 7390-81-0 | 23.079 | - | - | - | 0.7 | - | - |
| Naphthalene, 1,2,3,4-tetrahydro-1,6-dimethyl-4-(1-methylethyl)-, (1S-cis)- | 483-77-2 | 23.458 | - | - | 0.193 | 0.211 | - | - |
| Pentadecane, 3-methyl- | 2882-96-4 | 24.466 | 0.114 | - | - | - | - | - |
| Bicyclo[4.4.0]dec-1-ene, 2-isopropyl-5-methyl-9-methylene- | 150320-52-8 | 24.993 | - | - | - | - | 0.385 | - |
| Eicosane, 10-methyl- | 54833-23-7 | 25.138 | - | - | 0.218 | - | - | - |
| Hexadecane | 544-76-3 | 25.142 | 0.304 | - | - | - | - | 0.283 |
| Adamantane, 1-isothiocyanato-3,5-dimethyl- | 136860-49-6 | 26.564 | - | - | - | - | 0.451 | - |
| Heptadecane | 629-78-7 | 27.379 | 0.298 | 0.287 | 0.813 | - | 0.7 | 0.597 |
| 3-Amino-4,6-dimethylpyridone-2(1H) | 143708-29-6 | 8.971 | - | 0.444 | - | - | - | - |
| Octadecane | 593-45-3 | 29.409 | - | - | - | - | - | 0.113 |
| Tetracosane | 646-31-1 | 29.726 | - | - | 0.095 | - | - | - |
| Benzene, 1,1'-[1,2-ethanediylbis(oxy)]bis- | 104-66-5 | 29.95 | 0.626 | - | - | - | - | 0.293 |
| 3-Hexene, 2,5-dimethyl-3,4-bis(1-methylethyl)- | 7090-88-2 | 30.178 | 0.134 | - | - | - | - | - |
| 3,5-Diketo-1,6-heptadiene | 15849-12-4 | 30.186 | - | 2.324 | - | - | - | - |
| 1,3-Dioxolane, 2-(1,1-dimethylethyl)-2-methyl- | 6135-54-2 | 33.353 | - | - | - | - | - | 0.384 |
| **Phenols** |  |  | - | - | - | - | - | - |
| Phenol, 2-(1-methylpropyl)- | 89-72-5 | 18.184 | 0.479 | 0.201 | 0.279 | - | - | 0.475 |
| Phenol, 2,4,6-tris(1-methylethyl)- | 2934-07-8 | 22.947 | - | - | - | - | 0.391 | - |
| 4-(7-Methyloctyl)phenol | 24518-48-7 | 24.689 | - | - | - | - | 1.427 | - |
| 4a(2H)-Naphthalenol, 1,3,4,5,6,8a-hexahydro-4,7-dimethyl-1-(1-methylethyl)-, (1S,4R,4aS,8aR)- | 19912-67-5 | 25.332 | - | - | - | - | 2.412 | - |
| 4a(2H)-Naphthalenol, 1,3,4,5,6,8a-hexahydro-4,7-dimethyl-1-(1-methylethyl)-, (1S,4S,4aS,8aR)- | 73365-77-2 | 25.697 | - | - | - | - | 1.538 | - |
| 1-Naphthalenol, 1,2,3,4,4a,7,8,8a-octahydro-1,6-dimethyl-4-(1-methylethyl)-, [1R-(1.alpha.,4.beta.,4a.beta.,8a.beta.)]- | 19435-97-3 | 26.105 | - | - | - | - | 0.931 | - |
| **Other** |  |  | - | - | - | - | - | - |
| Anthracene, 9-ethyl-9,10-dihydro-9,10-dimethyl- | 54947-86-3 | 3.867 | - | - | - | 0.653 | - | - |
| Sarcosine ethyl ester hydrochloride | 52605-49-9 | 4.237 | - | - | - | - | - | 1.289 |
| l-Alanine ethylamide, (S)- | 71773-95-0 | 4.290 | - | 1.533 | - | - | - | - |
| 2-Propen-1-amine | 107-11-9 | 4.35 | - | - | - | 2.505 | - | - |
| Cyclopentanamine | 1003-03-8 | 4.378 | - | 11.574 | - | - | - | - |
| 1,2,4,5-Tetrazine | 290-96-0 | 4.386 | 0.777 | - | - | - | - | - |
| Propanoic acid, anhydride | 123-62-6 | 4.413 | - | - | 3.943 | - | - | - |
| Fomepizole | 7554-65-6 | 4.432 | - | 0.991 | - | - | - | - |
| 1-Propanamine, N-propyl- | 142-84-7 | 4.444 | - | - | - | 1.51 | - | - |
| Phenol, 4-[2-(methylamino)ethyl]- | 370-98-9 | 4.509 | 0.741 | - | - | - | - | - |
| 2-Aminocyanoacetamide | 6719-21-7 | 4.515 | 3.106 | - | - | 0.748 | - | - |
| 2-Propanone, hydrazone | 5281-20-9 | 4.518 | - | - | - | 0.262 | - | - |
| 3,4-Dimethoxycinnamic acid | 2316-26-9 | 4.538 | - | - | - | - | - | 0.325 |
| n-Hexylmethylamine | 35161-70-7 | 4.544 | - | 0.636 | - | - | - | - |
| m-Nitrobenzaldehyde dimethylhydrazone | 32787-76-1 | 4.776 | - | - | - | 0.113 | - | - |
| 1,3-Benzenedicarboxylic acid, 5-(1,1-dimethylethyl)- | 2359-09-3 | 4.926 | - | 0.115 | - | - | - | - |
| 4-(Anisylideneamino)-cinnamic acid | 25959-50-6 | 5.503 | - | 0.03 | - | 0.137 | - | - |
| (2-Aziridinylethyl)amine | 4025-37-0 | 6.552 | 0.287 | - | - | - | - | - |
| Sulfur dioxide | 7446-09-5 | 6.569 | 0.117 | - | - | - | - | - |
| 9-Thiabicyclo[3.3.1]non-6-en-2-amine, N-methyl-, endo- | 62545-67-9 | 6.572 | - | 0.636 | - | - | - | - |
| L-Alanine, 3-sulfo- | 498-40-8 | 6.577 | - | - | 1.008 | - | - | - |
| N-(9-Anthrylmethylene)aniline | 796-34-9 | 6.652 | - | 0.138 | - | 0.181 | - | - |
| 1-(1,2-Dimethoxypropyl)-4-methoxybenzene | 138169-72-9 | 6.674 | - | - | - | 0.615 | - | - |
| 2-Hydrazino-5-cyanopyridine | 104408-24-4 | 6.842 | - | - | 0.232 | - | - | - |
| (1H)Pyrrole-3-carbonitrile, 2-methyl- | 26187-27-9 | 8.174 | - | - | 0.503 | - | - | - |
| N-(3-Phenyl-1,2,4-thiadiazol-5-yl)benzamide | 17280-75-0 | 8.175 | - | - | 0.742 | - | - | - |
| 3-Pyridinecarboxamide, N-(5-nitro-2-thiazolyl)- | 64724-83-0 | 8.193 | - | 1.013 | - | - | - | - |
| 3-Amino-1-azabicyclo[2.2.2]octane | 6238-14-8 | 8.391 | - | - | 0.401 | - | - | - |
| Dimethyl trisulfide | 3658-80-8 | 8.418 | - | - | - | 0.662 | - | - |
| 1,3,5-Triazine | 290-87-9 | 8.609 | 0.516 | - | 0.227 | - | - | - |
| Propanoic acid, 2-methyl-, anhydride | 97-72-3 | 8.657 | - | - | 1.233 | - | - | - |
| 5-Amino-2-methyl-2H-tetrazole | 6154-04-7 | 8.659 | - | 2.493 | - | - | 1.48 | - |
| Furan, 2-[(methylthio)methyl]- | 1438-91-1 | 8.706 | - | - | - | - | - | 1.393 |
| 2,3,4,5-Tetrahydro-2-methyl-6-(2-(5-nitro-2-furyl)vinyl)-5-oxo-3-thioxo-1,2,4-triazine | 20113-58-0 | 8.77 | 0.126 | - | - | - | - | - |
| Pentanoic acid, 2-methyl-, anhydride | 63169-61-9 | 8.774 | - | - | 0.717 | 2.213 | - | - |
| 2-Acetyl-2-methyltetrahydrofuran | 32318-87-9 | 8.934 | - | 0.511 | - | - | - | - |
| Furan, 2-pentyl- | 3777-69-3 | 8.964 | 3.972 | - | - | - | - | - |
| 2-Ethylcyclohexanol,c&t | 3760-20-1 | 10.93 | - | - | - | - | - | 1.756 |
| 1H,5H,7H,11H-Dipyrazolo[1,2-a:1',2'-d][1,2,4,5]tetrazine, tetrahydro- | 37882-92-1 | 10.985 | - | - | - | - | 0.833 | - |
| Anthracene, 9-methoxy- | 2395-96-2 | 11.424 | - | - | - | - | - | 0.118 |
| 4,5-Dihydro-N-(O-tolyl)-3-furamide | 65038-88-2 | 11.524 | - | - | - | - | - | 0.631 |
| 1,3-Dioxolo[4,5-h]isoquinoline, 6,7,8,9-tetrahydro-4-methoxy-9-methyl-, (S)- | 519-04-0 | 11.542 | - | - | 0.026 | - | - | - |
| 1H-Pyrazole, 4,5-dihydro-4,5-dimethyl- | 28019-94-5 | 12.171 | - | 6.971 | - | - | - | - |
| Furan, 2-methoxy- | 25414-22-6 | 12.174 | - | - | - | 2.288 | - | - |
| 3,6-Bis(N,N-dimethylamino)-9-methylcarbazole | 119046-55-8 | 12.512 | - | 0.09 | - | - | - | - |
| Isoquinoline, 3,4-dihydro-6,7-dimethoxy-1-phenyl- | 10172-51-7 | 12.528 | - | - | 0.351 | - | - | - |
| Thiophene, 2-pentyl- | 4861-58-9 | 13.794 | - | - | 0.205 | - | - | - |
| 2-(1-Cyclopentenyl)furan | 115754-78-4 | 13.912 | - | - | - | 0.299 | - | - |
| Trimethylpyrazole-4-carbonitrile | 108161-13-3 | 13.913 | - | - | 0.199 | - | - | - |
| Undecane, 3-methyl- | 1002-43-3 | 14.057 | - | - | - | 0.307 | - | - |
| 1H-Pyrazole, 4,5-dihydro-3,5,5-trimethyl- | 3975-85-7 | 14.752 | - | - | 0.189 | - | - | - |
| Tetrasulfide, dimethyl | 5756-24-1 | 15.407 | 0.123 | 0.143 | 0.185 | 0.355 | - | - |
| Dihexyverine | 561-77-3 | 16.763 | 0.161 | - | - | - | - | - |
| 1H-Pyrazole, 4,5-dihydro-5-propyl- | 75011-90-4 | 16.771 | 0.152 | - | - | - | - | - |
| 5-Thiazoleethanol, 4-methyl- | 137-00-8 | 17.164 | 0.223 | - | - | - | - | - |
| N-(2-Methyl-2H-tetrazol-5-yl)-acetamide | 6154-06-9 | 17.252 | - | 0.354 | - | - | - | - |
| 2-n-Octylfuran | 4179-38-8 | 17.561 | - | 0.419 | 0.797 | 0.613 | 0.447 | 0.901 |
| Furan, 2-hexyl- | 3777-70-6 | 18.152 | - | 0.466 | - | - | 0.506 | - |
| Pyrazine, ethyl- | 13925-00-3 | 18.482 | - | - | - | - | 0.958 | - |
| (1S,4S,4aS)-1-Isopropyl-4,7-dimethyl-1,2,3,4,4a,5-hexahydronaphthalene | 267665-20-3 | 18.813 | - | - | - | - | 1.173 | - |
| Benzene, 1,4-diethoxy- | 122-95-2 | 18.965 | - | 0.144 | - | - | - | - |
| Uridine, 2'-deoxy-, 3',5'-diacetate | 13030-62-1 | 19.385 | 0.313 | - | - | - | - | - |
| Pilocarpine | 92-13-7 | 19.395 | - | 0.713 | - | - | - | - |
| Carbamodithioic acid, diethyl-, methyl ester | 686-07-7 | 19.878 | - | 0.097 | - | 0.227 | 0.364 | - |
| 1,1,7,7a-Tetramethyl-1a,2,6,7,7a,7b-hexahydro-1H-cyclopropa[a]naphthalene | 154098-14-3 | 20.301 | - | - | - | - | 0.524 | - |
| 2-Propen-1-amine, N-2-propenyl- | 124-02-7 | 20.537 | - | - | 0.166 | - | - | - |
| (3R,3aS,8aS)-3,6,8,8-Tetramethyl-2,3,4,7,8,8a-hexahydro-1H-3a,7-methanoazulene | 22567-43-7 | 20.749 | - | - | 0.113 | - | - | - |
| Naphthalene, 1,5-dimethyl- | 571-61-9 | 20.846 | - | 0.092 | - | - | - | - |
| (+)-epi-Bicyclosesquiphellandrene | 54274-73-6 | 21.76 | - | - | - | - | 1.497 | - |
| 1-Butanamine, N-(2-pyridinylmethylene)- | 7032-24-8 | 21.868 | - | - | 0.318 | - | - | - |
| N-Benzyl-2-phenethylamine | 3647-71-0 | 21.869 | 0.299 | - | - | - | - | - |
| 1H-Imidazo[4,5-b]pyridine | 273-21-2 | 21.872 | 0.271 | - | 0.272 | - | - | - |
| 2,5-di-tert-Butyl-1,4-benzoquinone | 2460-77-7 | 22.159 | 0.145 | - | - | - | - | - |
| p-Aminotoluene | 106-49-0 | 22.206 | - | - | - | - | 0.618 | - |
| trans-.alpha.-Bergamotene | 13474-59-4 | 22.463 | - | - | - | - | 1.574 | - |
| Benzene, 1,2,4,5-tetraethyl- | 635-81-4 | 22.554 | - | - | - | - | 1.21 | - |
| N-Ethyl-2-methyl-5-benzimidazolecarboxamide | 62306-07-4 | 22.652 | - | - | - | - | 0.45 | - |
| Naphthalene, 1,2,3,4,4a,5,6,8a-octahydro-7-methyl-4-methylene-1-(1-methylethyl)-, (1.alpha.,4a.beta.,8a.alpha.)- | 39029-41-9 | 23.006 | - | - | - | - | 4.744 | - |
| Methyl-(9-oxa-bicyclo[3.3.1]non-6-en-2-yl)-amine | 63827-19-0 | 23.070 | - | 0.142 | - | - | - | - |
| Butylated Hydroxytoluene | 128-37-0 | 23.172 | 0.249 | - | - | - | - | - |
| 2H-1-Benzopyran, 6,7-dimethoxy-2,2-dimethyl- | 644-06-4 | 23.172 | 0.234 | - | - | - | - | - |
| trans-Calamenene | 73209-42-4 | 23.456 | 0.118 | 0.147 | 0.2 | - | - | - |
| Cyclohexane, 1,2-dimethyl-3,5-bis(1-methylethenyl)- | 62337-99-9 | 24.627 | - | - | - | - | 1.242 | - |
| 4,7(1H,8H)-Pteridinedione | 33669-70-4 | 24.629 | - | - | - | - | 0.625 | - |
| 3,5-Dimethyl-4-propyl-1H-pyrazole | 81328-51-0 | 25.286 | - | - | - | - | 0.414 | - |
| .alpha.-Furil | 492-94-4 | 25.786 | - | - | - | - | 0.505 | - |
| Benzo[1,2-c:3,4-c':5,6-c'']tris[1,2,5]oxadiazole | 16279-15-5 | 26.002 | - | - | - | - | 1.513 | - |
| Acrylanilide | 2210-24-4 | 26.016 | - | 0.39 | - | - | 0.867 | - |
| Pyridinium, 1-(acetylamino)-, hydroxide, inner salt | 1468-29-7 | 26.021 | - | 0.367 | - | - | 2.234 | - |
| Naphthalene, 1,6-dimethyl-4-(1-methylethyl)- | 483-78-3 | 26.761 | - | - | - | - | 0.676 | - |
| 1,1'-Biphenyl, 2,2',5,5'-tetramethyl- | 3075-84-1 | 27.038 | 0.111 | - | - | - | - | - |
| 1H-Pyrrole-2,5-dione, 1-ethyl- | 128-53-0 | 27.718 | - | 0.176 | - | - | - | - |
| 3-Ethoxyacrylonitrile | 61310-53-0 | 27.723 | - | - | 0.422 | - | - | - |
| 3,3-Tetramethyleneglutaric anhydride | 5662-95-3 | 27.725 | - | - | 0.469 | - | - | - |
| (1S,3aR,4R,8R,8aS)-1-Isopropyl-3a-methyl-7-methylenedecahydro-4,8-epithioazulene | 72445-42-2 | 28.147 | - | - | - | - | 0.822 | - |
| Benzenamine, N,N-dimethyl- | 121-69-7 | 29.948 | 0.272 | - | - | - | - | - |
| 2-Pyrazoline, 5-ethyl-1,4-dimethyl- | 14339-23-2 | 30.18 | - | - | - | 0.601 | - | - |
| n-Hexadecanoic acid | 57-10-3 | 34.668 | - | - | 0.086 | - | - | - |

Note: RI is the retention time
